# Neuroprotective effects of flavonoids: endoplasmic reticulum as the target

**DOI:** 10.3389/fnins.2024.1348151

**Published:** 2024-06-18

**Authors:** Bita Amiri, Maryam Yazdani Tabrizi, Mahdyieh Naziri, Farzaneh Moradi, Mohammadreza Arzaghi, Iman Archin, Fatemeh Behaein, Anahid Bagheri Pour, Parna Ghannadikhosh, Saba Imanparvar, Ata Akhtari Kohneshahri, Ali Sanaye Abbasi, Nasibeh Zerangian, Dorsa Alijanzadeh, Hani Ghayyem, Arash Azizinezhad, Mahya Ahmadpour Youshanlui, Mohadeseh Poudineh

**Affiliations:** ^1^Cardiovascular Research Center, Tabriz University of Medical Sciences, Tabriz, Iran; ^2^Student Research Committee, School of Medicine, Shahid Beheshti University of Medical Sciences, Tehran, Iran; ^3^Student Research Committee, School of Health, Iran University of Medical Sciences, Tehran, Iran; ^4^Department of Physical Education and Sports Science-Nutrition, Branch Islamic Azad University, Tehran, Iran; ^5^Shahid Beheshti University of Medical Sciences, Tehran, Iran; ^6^Kazan (Volga Region) Federal University, Kazan, Russia; ^7^Iran University of Medical Sciences, Tehran, Iran; ^8^Student Research Committee, Tabriz University of Medical Sciences, Tabriz, Iran; ^9^School of Medicine, Ardabil University of Medical Sciences, Ardabil, Iran; ^10^Student Research Committee, Faculty of Medicine, Tabriz Medical Sciences, Islamic Azad University, Tabriz, Iran; ^11^Student Research Committee, School of Medicine, Guilan University of Medical Sciences, Rasht, Iran; ^12^PhD Student in Health Education and Health Promotion, Department of Health Education and Health Promotion, School of Health, Mashhad University of Medical Sciences, Mashhad, Iran; ^13^School of Medicine, Shahid Beheshti University of Medical Sciences, Tehran, Iran; ^14^Universal Scientific Education and Research Network (USERN), Tabriz, Iran; ^15^Faculty of Medicine, Tabriz University of Medical Sciences, Tabriz, Iran; ^16^Student Research Committee, Zanjan University of Medical Sciences, Zanjan, Iran

**Keywords:** ZUMS, Gavazang road, Zanjan, Iran flavonoids, endoplasmic reticulum stress, neurological disease, Alzheimer’s disease, Parkinson’s disease

## Abstract

The incidence of neurological disorders, particularly age-related neurodegenerative pathologies, exhibits an alarming upward trend, while current pharmacological interventions seldom achieve curative outcomes. Despite their diverse clinical presentations, neurological diseases often share a common pathological thread: the aberrant accumulation of misfolded proteins within the endoplasmic reticulum (ER). This phenomenon, known as ER stress, arises when the cell’s intrinsic quality control mechanisms fail to cope with the protein-folding burden. Consequently, misfolded proteins accumulate in the ER lumen, triggering a cascade of cellular stress responses. Recognizing this challenge, researchers have intensified their efforts over the past two decades to explore natural compounds that could potentially slow or even reverse these devastating pathologies. Flavonoids constitute a vast and heterogeneous class of plant polyphenols, with over 10,000 identified from diverse natural sources such as wines, vegetables, medicinal plants, and organic products. Flavonoids are generally divided into six different subclasses: anthocyanidins, flavanones, flavones, flavonols, isoflavones, and flavonols. The diverse family of flavonoids, featuring a common phenolic ring backbone adorned with varying hydroxyl groups and additional modifications, exerts its antioxidant activity by inhibiting the formation of ROS, as evidenced by research. Also, studies suggest that polyphenols such as flavonoids can regulate ER stress through apoptosis and autophagy. By understanding these mechanisms, we can unlock the potential of flavonoids as novel therapeutic agents for neurodegenerative disorders. Therefore, this review critically examines the literature exploring the modulatory effects of flavonoids on various steps of the ER stress in neurological disorders.

## 1 Introduction

Flavonoids, a diverse group of polyphenols naturally found in fruits, vegetables, coffee, and wine, transcend their well-known anti-inflammatory, antioxidant, and antitumor properties (Panche et al., 2016; Talebi et al., 2021). Recent research highlights their remarkable ability to modulate the activity of key enzymes implicated in various disease processes. Studies demonstrate their inhibitory potential against diverse targets including COX, lipoxygenase, Ca2+ ATPase, xanthine oxidase, aldose reductase, and phosphodiesterase, suggesting their potential application across a spectrum of pathological conditions (Panche et al., 2016). Classified based on their C-ring structure, they encompass diverse subgroups such as flavonols, flavones, flavanones, neoflavonoids, isoflavones, anthocyanidins, flavanonols, chalcones, and flavanols/catechins (Justesen and Knuthsen, 2001).

### 1.1 Flavonols

Flavonols represent a distinct subclass of flavonoids characterized by the presence of a ketone group. This group encompasses widely studied members like kaempferol, quercetin, rutin, myricetin, and fisetin. Evidence suggests a positive association between flavonol intake and various health benefits, particularly attributed to their potent antioxidant activity. For instance, quercetin is found in abundance across diverse fruits, vegetables, beverages, and spices, contributing to overall dietary intake. Moreover, Strawberries, apples, persimmons, onions, and cucumbers are good sources of Fisetin (Stewart et al., 2000; Zheng and Wang, 2001; Robertson and Nichols, 2017). Notably, George Robertson and Matthew Nichols have demonstrated the ability of compositions containing a flavan-3-ol (e.g., epicatechin), a flavonoid (e.g., quercetin), and a fatty acid (e.g., EPA ethyl ester) to mitigate oxidative damage associated with mitochondrial dysfunctions (Author, 2013). This work suggests promising avenues for treating various neurological disorders such as Parkinson’s, Huntington’s, amyotrophic lateral sclerosis (ALS), Alzheimer’s, and multiple sclerosis (MS), potentially extending to neuroprotection against stroke-induced damage and cisplatin-induced ototoxicity.

This invention, attributed to Amalia Porta, focuses on synthetic and plant-derived flavonoid compounds represented by formulas (I) and (II). These compounds exhibit the unique ability to modulate the dynamic and physical state of biological membranes within eukaryotic cells. Additionally, they stimulate the endogenous synthesis of stress proteins, offering potential therapeutic implications. The invention provides a comprehensive methodology for the identification, purification, and chemical synthesis of these specific flavonoids. Further, it outlines a testing strategy that evaluates their efficacy through their capacity to induce stress gene transcription and their subsequent interaction with biological membranes, ultimately altering their physical characteristics. Beyond their potential use in the pharmaceutical industry, these compounds and their pharmaceutically acceptable derivatives/salts hold promise within the field of cosmetics and dermatology. Specifically, they may provide therapeutic approaches for addressing conditions associated with altered stress gene expression (Shimoi et al., 1998).

### 1.2 Flavones

Flavones, a subclass of flavonoids, encompass widely studied members like luteolin, apigenin, and tangeritin. These compounds occur abundantly in various parts of plants, including leaves, flowers, and fruits, contributing significantly to dietary intake. For instance, luteolin can be readily extracted from a diverse range of plant sources, including broccoli, green pepper, celery, parsley, thyme, dandelion, tea, carrots, olive oil, peppermint, and rosemary. Additionally, the peels of citrus fruits serve as a rich reservoir of flavones, contributing to their characteristic flavors and potential health benefits (Khan et al., 2009; López-Lázaro, 2009).

### 1.3 Flavanones

Flavanones, a subgroup of flavonoids, include renowned members like hesperidin, naringenin, and eriodictyol. These compounds are recognized for their free radical scavenging abilities, contributing to various health-promoting effects. Specifically, they exhibit anti-inflammatory, antioxidant, and blood lipid-lowering properties, highlighting their potential therapeutic applications. Interestingly, flavanones are responsible for the characteristic bitter taste found in the juice and peel of citrus fruits. Grapes and citrus fruits, particularly oranges and lemons, serve as excellent sources of these beneficial compounds (Felgines et al., 2000; Donnelly and Neoflavonoids, 2017).

### 1.4 Neoflavonoids

They are polyphenolic compounds and have shown widespread distribution. They have shown anti-osteoporosis, anti-inflammatory, antitumor, anti-allergic, and antioxidation qualities (Iinuma et al., 1987; Aoki et al., 2000; NISHIMUTA et al., 2000; Garazd et al., 2003).

### 1.5 Isoflavonoids

Despite its large size, isoflavonoids exhibit a limited natural distribution, primarily found in legumes like soybeans and some microbial sources. Notable members include genistein and daidzein, two isoflavonoids garnering substantial scientific interest due to their potential health benefits. Genistein, in particular, has been associated with preventative effects against various chronic conditions. Studies suggest its potential in reducing the risk of breast and prostate cancer, mitigating post-menopausal symptoms like hot flashes, and contributing to cardiovascular health by improving cholesterol profiles and reducing inflammation (Dixon and Ferreira, 2002; Szkudelska and Nogowski, 2007; Mattioli et al., 2020).

Anthocyanins, a captivating subclass of water-soluble flavonoids, adorn fruits and vegetables with their diverse colors. Prominent members include cyanidin, delphinidin, pelargonidin, petunidin, and peonidin, showcasing an interplay between their structure and their vibrant hues depending on pH. Beyond their esthetic appeal, these pigments constitute the primary source of color in plants. Intriguingly, the significance of anthocyanins extends far beyond mere esthetics. Extensive research delves into their potential health benefits, encompassing diverse physiological systems. Studies suggest that anthocyanins can modulate the circulatory, nervous, digestive, urinary, sensory, endocrine, and immune systems (Elias et al., 1999; Higdon and Frei, 2003; Liu et al., 2021). These promising bioactivities have fueled their exploration as dietary supplements, particularly for promoting eye health.

### 1.6 Chalcones

Some examples of this subclass are phloridzin, arbutin, phloretin, and chalconaringenin. They are found in numerous fruits and vegetables like tomatoes, pears, strawberries, and wheat products. They have a lot of nutritional and health-promoting benefits like cytotoxic, anticancer, and chemopreventative effects (Justesen and Knuthsen, 2001; Anandh Babu and Liu, 2008).

### 1.7 Flavanols or catechins

They are present in different fruits like apples, bananas, peaches, blueberries, and pears. Catechins contribute to the beneficial effects of tea. Tea catechins show antioxidant activity by scavenging free radicals. Catechins prevent the progression of atherosclerotic lesions by limiting vascular inflammation (Tham et al., 1998; Tikkanen and Adlercreutz, 2000).

Flavonoids offer a remarkable defense against free radical-mediated damage within the human body. Several intricate mechanisms contribute to this protective effect, with direct scavenging of free radicals being a prominent strategy. When flavonoids encounter these reactive oxygen species (ROS), they readily donate electrons, stabilizing the harmful radical and neutralizing its damaging potential. This process leads to the oxidation of the flavonoid itself, but the resulting molecule is typically much less reactive, minimizing further cell damage. Intriguingly, research suggests that all subclasses of flavonoids possess some degree of antioxidant activity, highlighting their broad-spectrum protective capabilities. However, specific subclasses demonstrate particularly potent effects. Studies indicate that flavonols and catechins stand out as powerful free radical scavengers, offering promising avenues for therapeutic interventions aimed at mitigating oxidative stress and its associated pathologies (Justesen and Knuthsen, 2001).

Flavonoids they exhibit protective effects against atherosclerosis, a major risk factor for cardiovascular diseases. Studies suggest their ability to modulate key parameters like cardiovascular reactivity, lipoprotein oxidation, and blood platelet aggregation, thereby reducing the risk of plaque formation and subsequent complications (Ren et al., 2003; Cushnie and Lamb, 2005). Flavonoids also have demonstrated antimicrobial activities and are considered possible antimicrobial agents or groups of agents (Fotsis et al., 1997). Additionally, flavonoids display promising anticancer properties. Research suggests their chemopreventive effects, potentially inhibiting the initiation and progression of cancer. Moreover, they can induce apoptosis in cancer cells through various mechanisms, including cell cycle arrest, modulation of oncogene expression, and regulation of carcinogen metabolism (Si and Liu, 2007; Kamaraj et al., 2009; Huang et al., 2010; Jellinger, 2010; Roussel et al., 2013; Feigin et al., 2019).

ER serves as a vital cellular hub for protein folding, maturation, and secretion. When this intricate process falters, a stress response termed ER stress ensues. This can trigger downstream pathways leading to apoptosis (programmed cell death), neuronal degeneration, and, ultimately, neurodegenerative diseases like Parkinson’s and Alzheimer’s disease (AD) (Roussel et al., 2013). While the connection between ER stress and these pathologies is increasingly recognized, a deeper understanding of the precise mechanisms involved and the specific proteins playing critical roles remains an active area of research (Jellinger, 2010).

The escalating burden of neurological disease worldwide demands innovative therapeutic strategies. Over the past 30 years, disability-adjusted life years (DALYs) associated with neurological disorders have risen by 15%, with a 39% increase in absolute mortality. This burden disproportionately affects middle-and low-income countries, despite diminishing rates of communicable neurological disorders (Feigin et al., 2019). The significant limitations of current treatment options for dementia and other neurodegenerative diseases fuel the exploration of novel therapies, including flavonoids. A promising avenue lies in the development of new flavonoid-based drugs using molecular docking techniques (Perez-Vizcaino and Fraga, 2018). Notably, specific flavonoids, such as those found in citrus fruits, demonstrate the ability to cross the blood–brain barrier (BBB) and exert diverse effects on the central nervous system (CNS). These include anxiolytic, anticonvulsant, and antidepressant effects, along with the potential to reverse ischemic reperfusion injury and improve symptoms of Parkinson’s disease (PD) (Roohbakhsh et al., 2014; Goyal et al., 2022, 2023). While the precise mechanisms of neuronal death in PD remain incompletely understood, neuroinflammation, ER stress, and impaired protein degradation are recognized contributors (Antony et al., 2013). This review explores the neuroprotective properties of various flavonoid classes and their potential role in the pharmacological treatment of neurological diseases, particularly through modulating ER function.

## 2 Methods

In order to find all articles relevant to the neuroprotective effects of flavonoids, the endoplasmic reticulum is the target. We applied the EMBASE, google scholar, web of science, research gate, and PubMed up to September 2022. No restrictions were applied to the language of the articles. We included Mesh terms and used an independent search schedule design for each database. Our search strategy was as follows:

#1 Flavonoid OR methoxy flavone OR flavanol OR Baicalein OR Proanthocyanidin OR luteolin OR Neohesperidin OR quercetin OR Troxerutin OR liquiritigenin OR Rutin OR kaempferol OR isorhamnetin OR Fisetin OR morin OR flavanones OR hesperidin OR naringenin OR eriodictyol OR flavones OR apigenin OR luteolin OR tangerine OR chrysin OR wogonin OR diosmin OR baicalein OR isoflavones OR genistein OR daidzein OR Pelargonidin OR anthocyanins OR cyanidin OR delphinidin OR bioflavonoid OR pelargonidin.

#2 neuron OR “Nerve Cell” OR “glial cell” OR Glia OR neuroglia OR brain OR Cerebral OR encephalon OR “spinal cord” OR neurological OR neurology OR neural OR neuronal OR Neuroprotective OR “Nervous System Disorder” OR neurodegenerative OR “Nerve Damage” OR “brain injury” OR “Nervous System Neoplasm” OR “Nervous System Malformation” OR demyelinating OR encephalopathy OR neuroinflammation OR neurotoxicity OR “Spinal Cord Disease” OR “age-related neurological disorder” OR “Myasthenia Gravis” OR Myelopathy OR encephalitis OR encephalomyelitis OR meningitis OR neuropathy OR neuroma OR “Alzheimer’s Disease” OR “Huntington’s Disease” OR “Multiple sclerosis” OR “Parkinson’s Disease” OR stroke OR paralysis OR chorea OR “Amyotrophic Lateral Sclerosis” OR ataxia OR “Brain Tumor” OR Seizures OR anorexia OR bulimia OR migraine OR sciatica OR headache OR dementia OR Glioma OR glioblastoma OR oligodendroglioma OR oligoastrocytoma OR GBM OR ANDs OR epilepsy OR RAS OR Atherosclerotic OR gliosarcoma OR neuroblastoma OR “Hereditary Spastic paraplegia” OR Fibromyalgia OR “Polyglutamine disease” OR “cognitive deficit” OR “diabetic neuropathy” OR “cognitive deficit” OR “impaired memory” OR “neuronal cell death” OR Dendrite OR cerebrum OR Axon OR astrocyte OR synapse OR interneuron OR neuromuscular OR neurocutaneous OR myelin.

#"Endoplasmic Reticulum.”

#4 #1 AND #2 AND #3.

All observational studies on the neuroprotective effects of flavonoids were included focusing on the endoplasmic reticulum as the target. Moreover, we kept articles focusing on the neuroprotective effects of flavonoids on endoplasmic reticulum stress. We excluded similar and identical studies and screened the abstract and title of the studies. After we acquired the full texts, the reference sections were reviewed to recognize extra research.

### 2.1 The effect of flavonoids on ER stress in neurological disorders

#### 2.1.1 Glioblastoma/Glioma

Disrupting the Nanog/Sox2/CD133 pathway, a key driver of glioma stemness, presents a promising therapeutic strategy for gliomagenesis inhibition. Potential targets within this pathway include inhibitors of miR-26a or AP-2, offering avenues for halting the self-renewal and tumorigenic potential of glioma stem cells. Furthermore, restoring p53 function, a potent tumor suppressor often compromised in glioblastoma (GBM), emerges as another attractive approach. Disrupting the p53/MDM2 interaction using molecules like ISA27 and nutlin-3a, or employing p53-binding circular RNAs (e.g., CDR1as), can effectively reinstate p53 activity and trigger apoptosis in undifferentiated GBM cells, thereby impeding tumor progression (Esemen et al., 2022).

Moreover, Santos et al. (2015) conducted a comprehensive investigation into the effects of various polyhydroxylated flavonoids such as rutin, quercetin, apigenin, chrysin, kaempferol, and 3′,4′-dihydroxyflavone. Their study revealed promising anticancer activities of these compounds against human glioblastoma cells. Notably, the study observed that these flavonoids induced differentiation, inhibited migration and invasive activity, and triggered apoptosis in glioblastoma cells. Interestingly, the apoptotic mechanism involved destruction of the rough ER and mitochondria, highlighting a unique mode of action for these flavonoids. Additionally, the study demonstrated that flavonoids effectively inhibited cell migration by decreasing the number of filopodia-like structures on the cell surface. This effect was attributed to downregulation of Matrix metalloproteinase-2 (MMP-2) activity and expression, coupled with increased intracellular fibronectin and laminin expression, suggesting modulation of the extracellular matrix as another facet of their anticancer mechanism (Santos et al., 2015).

#### 2.1.2 Quercetin

Jang et al. (2020) investigated the synergistic effects of combining chloroquine, a lysosome-targeting antimalarial, with the flavonoid quercetin in malignant glioma cells. This study revealed a novel caspase-independent apoptotic pathway triggered by this combination. The key mechanism involved the excessive expansion of lysosomes and autolysosomes, overwhelmed by accumulating protein aggregates and undigested cellular components, ultimately leading to cell death. Furthermore, the study elucidated a complex interplay between intracellular calcium signaling and reactive oxygen species (ROS) generation in this synergistic cytotoxicity. Specifically, the authors demonstrated that the combination induced 1,4,5-triphosphate receptor (IP3R)-mediated Ca2+ release from the ER, followed by ROS generation and subsequent mitochondrial Ca2+ influx through the mitochondrial uniporter (MCU). This intricate calcium signaling cascade ultimately contributed to mitochondrial dysfunction and cell death. Therefore, this study highlights the potential of the quercetin and chloroquine combination as an effective therapeutic strategy for glioblastoma (GBM) by inducing a unique, caspase-independent apoptotic pathway mediated by lysosome overload and calcium signaling dysregulation. Further research is warranted to explore the clinical efficacy and safety of this promising combination therapy in GBM treatment.

#### 2.1.3 Wogonin

Tsai et al. (2012) investigated the anti-cancer potential of wogonin, a flavonoid compound, in human glioma cell lines U251 and U87. Their study elucidated a novel mechanism of wogonin-induced apoptosis involving ER stress and ROS generation. The analysis revealed that wogonin treatment effectively triggered cell death in both glioma cell lines. Mechanistically, this effect was mediated by the activation of caspase-3 and caspase-9, key executioners in the apoptotic pathway, along with an upregulation of cleaved PARP, a marker of DNA fragmentation. Further investigations revealed enhanced expression of several ER stress markers, including GRP78, GRP94, and phosphorylated eIF2α, suggesting wogonin’s ability to induce ER stress in glioma cells. Additionally, the study demonstrated an increase in ROS generation upon wogonin treatment, highlighting a potential contribution to its cytotoxic effects. In conclusion, this study suggests that wogonin exhibits promising anti-cancer activity against human glioma cells by inducing apoptosis through a dual mechanism involving ER stress activation and ROS generation. Further research is warranted to explore the therapeutic potential of wogonin as a complementary or alternative approach in glioma management.

#### 2.1.4 Luteolin

Wang et al. (2017) delved into the molecular mechanisms underlying the anticancer effects of luteolin, a flavonoid, in GBM cell lines U251MG and U87MG. Their study demonstrated that luteolin treatment effectively triggers apoptosis in these GBM cells. Furthermore, *in vivo* experiments revealed that luteolin administration significantly inhibits tumor xenograft growth in U87MG models. A key finding of the study was the identification of ROS accumulation as a pivotal mediator of luteolin’s action. This ROS induction, in turn, led to severe ER stress, evidenced by elevated levels of ER stress-associated proteins such as phosphorylated PERK, eIF2α, ATF4, CHOP, and cleaved-caspase-12. These changes ultimately culminated in mitochondrial dysfunction and subsequent apoptosis. Notably, pretreatment with the antioxidant N-acetylcysteine reversed ROS production, effectively abrogating luteolin-induced ER stress, mitochondrial dysfunction, and apoptosis, further solidifying the crucial role of ROS in this pathway.

#### 2.1.5 Apigenin

Das et al. (2010) explored the pro-apoptotic effects of various flavonoids on human GBM cell lines U87MG and T98G. Treatment with apigenin (APG), epigallocatechin (EGC), epigallocatechin-3-gallate (EGCG), and genistein (GST) effectively induced apoptosis in both cell lines, evident by morphological and biochemical changes. Mechanistically, these flavonoids increased dichlorofluorescein (DCF) oxidation, suggesting their ability to elevate intracellular ROS levels and activate p38 Mitogen-activated protein kinases (MAPKs). Subsequently, c-Jun N-terminal Protein Kinase 1 (JNK1) was activated, leading to the transcription of pro-apoptotic genes. Additionally, flavonoids suppressed the Akt and B-cell lymphoma 2 (Bcl-2) survival pathways, while also downregulating nuclear factor-kappa B (NF-κB) and cyclooxygenase-2 (COX-2) expression. Furthermore, EGC and EGCG treatment specifically induced the mitochondrial apoptosis pathway, as evidenced by increased active caspase-8, proteolytic cleavage of Bid, and Bax upregulation with Bcl-2 downregulation at both mRNA and protein levels. This pathway involved the loss of mitochondrial membrane potential (ΔΨm), release of cytochrome c and Smac into the cytosol, and activation of caspase-9. Finally, the study demonstrated that flavonoids elevated intracellular free calcium ([Ca2+]), leading to the activation of calpain, caspases-3 and-4, and ER stress, collectively contributing to apoptosis induction in GBM cells.

### 2.2 Neurotoxicity

#### 2.2.1 Alpha-naphthoflavone

Yu et al. (1999) (10, 11 delved into the molecular mechanisms) underlying the cytotoxicity and apoptosis induced by alpha-naphthoflavone (aNF) in mouse HT22 hippocampal cells. Their study revealed that aNF triggers apoptotic cell death through a multi-pronged approach. One key mechanism involved ER stress, evidenced by increased expression of proteins like C/EBP homologous protein and activation of caspases 12 and 3. Interestingly, the authors successfully mitigated aNF-induced ER stress using salubrinal (eIF2α phosphatase inhibitor) and CHOP siRNA transfection, highlighting its crucial role in the apoptotic pathway. Furthermore, the study implicated Mitogen-activated protein kinases (MAPKs), specifically ERK, JNK, and p38, in ROS accumulation, which further contributed to cytotoxicity. The antioxidant N-acetyl-cysteine effectively diminished aNF-induced CHOP expression, suggesting a link between ROS and ER stress activation. Another critical player identified was c-Src kinase, activated by aNF. Inhibition of c-Src using the kinase inhibitor SU6656 or siRNA transfection, led to reduced AhR transcriptional activity induced by aNF. This suggests a c-Src-MAPK-AhR signaling axis contributing to aNF’s cytotoxic effects. In conclusion, this study unraveled a multifaceted mechanism of aNF-induced cytotoxicity and apoptosis in hippocampal cells, involving ER stress, ROS generation, MAPK activation, and c-Src signaling. These findings shed light on potential therapeutic targets for mitigating aNF toxicity.

#### 2.2.2 Methoxy flavone

Takano et al. (2007) investigated the protective effects of methoxyflavones, a class of flavonoids, against ER stress in mouse cells. Their findings revealed that methoxyflavones mildly activate the Nrf2 pathway and eukaryotic initiation factor 2 (eIF2) pathway, leading to the induction of downstream genes like glucose-regulated protein (GRP). Interestingly, the protein kinase A (PKA) pathway played a critical role in these effects. Studies showed that agents like IBMX, dibutyryl-cAMP, and forskolin, which elevate intracellular cAMP levels, enhanced the protective effects of methoxyflavones, while the PKA inhibitor H^−89^ suppressed them. This suggests that the PKA pathway is instrumental in methoxyflavone-mediated ER stress modulation. Further *in vivo* experiments demonstrated the remarkable neuroprotective potential of methoxyflavones. Pre-administration of tangerine, a specific methoxyflavone, in mice increased the expression of HO-1 and GRP78 in the substantia nigra pars compacta, but without inducing ER stress itself. Moreover, it inhibited tunicamycin-induced cell death and protected dopaminergic neurons from the neurotoxin 1-methyl-4-phenyl-1,2,3,6-tetrahydropyridine (MPTP), which triggers both oxidative and ER stress. These findings collectively paint a promising picture for methoxyflavones as potential therapeutic agents for ER stress-related diseases, particularly neurodegenerative disorders.

### 2.3 Neuroblastoma

The delayed activation or modification of typical apoptotic pathways may be significant in spontaneous regression and treatment resistance of neuroblastoma. The Bcl-2 family, survivin, and caspase-8 are significant components of the apoptotic signaling cascade with aberrant expression or activation patterns (Castle et al., 1993; Hopkins-Donaldson et al., 2000; Teitz et al., 2000; Eggert et al., 2001). Epigenetic silencing results in inactivation that primarily affects the latter. The hypermethylation of CpG islands in the promoters of genes frequently causes gene functional inactivation. This way of inactivation for caspase-8, the four TRAIL apoptosis receptors, the RASSF1A tumor suppressor, the caspase-8 inhibitor FLIP, p73, DAPK, RB1, p14ARF, CD44, and p16INK4a, has been observed in neuroblastoma (Eggert et al., 2001; Van Noesel et al., 2002).

#### 2.3.1 Kaempferol

**Figure 1:**
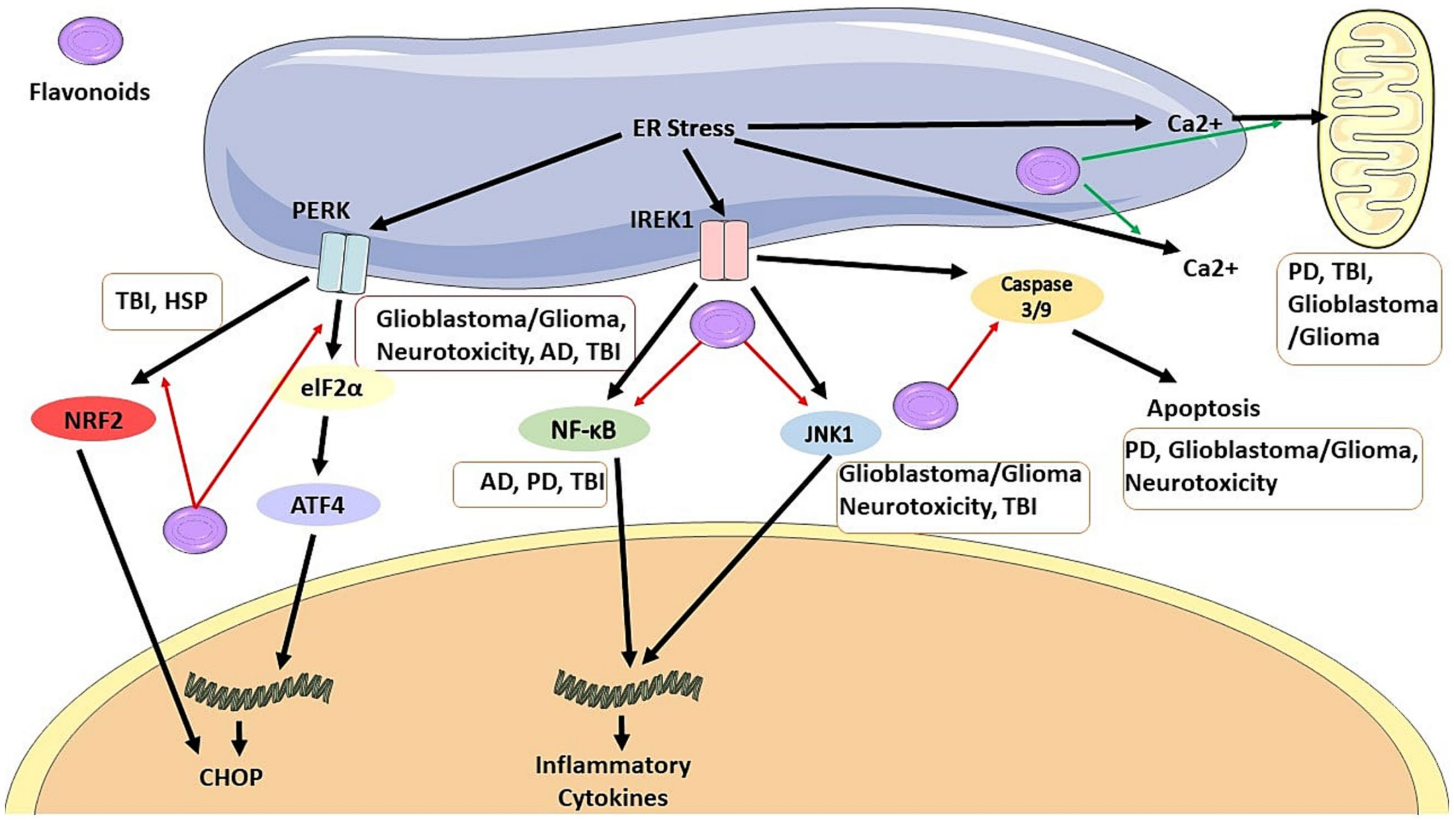
Flavonoids can inhibit CHOP pathway by blocking NRF2 signaling pathway in TBI and HSP, and by blocking elF2a/ATF4 pathway in glioblastoma/glioma, neurotoxicity, AD, and TBI. It also can inhibit production of inflammatory cytokines by blocking NF-KB pathway in AD, PD, and TBI, and also by blocking JNK1 pathway in glioblastoma/glioma, neurotoxicity, and TBI. Flavonoids has anti-apoptosis effects by inhibiting caspase 3/9 production in PD, glioblastoma/ glioma, and neurotoxicity. It also regulate Ca2+ in PD, TBI, and glioblastoma/glioma.

Neuroblastoma is the foremost prevalent cancer with a neural crest origin known to regress through cell differentiation by operators such as alpha trans-retinoic acid (ATRA) (Matthay et al., 1999). Phytoestrogen Kaempferol is a member of the flavonoids family and shows antioxidant, anti-inflammatory, and anticancer effects. Allosteric inhibition of killer caspases can block ER stress-induced cell damage and apoptosis (Abdullah and Ravanan, 2018a; Wang et al., 2018). Inositol-requiring enzyme one alpha (IRE1α) is an ER membrane protein enacted by misfolded proteins or other boosts and initiates cell differentiation through the IRE1α-XBP1 pathway (Reimold et al., 2001; Abdullah and Ravanan, 2018b). In this regard, the treatment of neuroblastoma cells with either ATRA or Kaempferol was investigated by Abdullah et al. (2019). Their study revealed a promising avenue for harnessing kaempferol’s ability to modulate the integrated stress response protein IRE1α to induce apoptosis and differentiation in these cancer cells. Treatment with kaempferol resulted in enhanced IRE1α activity, leading to an increase in cell death and increased expression of neuronal markers, a well-recognized hallmark of successful neuroblastoma differentiation therapy. These findings suggest that kaempferol possesses promising anti-tumorigenic potential by promoting both apoptosis and differentiation in neuroblastoma cells (Abdullah et al., 2019; Figure 1).

#### 2.3.2 Neohesperidin

Ogura et al. (2021) investigated the link between glucose-induced oxidative stress and protein dysfunction in neuroblastoma cells. Their study revealed that high glucose levels trigger protein S-nitrosylation, a process potentially contributing to neurodegenerative pathologies like AD. Notably, S-nitrosylation was also observed in brain tissues of AD patients, highlighting its potential relevance to disease development. Focusing on potential therapeutic interventions, the study identified neohesperidin, a natural flavonoid found in citrus fruits, as a promising candidate. This potent antioxidant exhibited remarkable neuroprotective effects. When administered to neuroblastoma cells during the final seven days of high glucose exposure, neohesperidin effectively reversed protein S-nitrosylation and significantly increased cell proliferation, suggesting its ability to mitigate the detrimental effects of oxidative stress.

#### 2.3.3 Quercetin

Chakraborty et al. investigated the neuroprotective potential of quercetin against copper (Cu)-induced apoptosis in SH-SY5Y neuroblastoma cells. Their study revealed that Cu exposure triggers excessive ROS generation, leading to morphological alterations and nuclear condensation in these cells. Additionally, Cu disrupts the mitochondrial membrane potential, ultimately culminating in apoptotic cell death. Interestingly, quercetin was shown to effectively reverse these deleterious effects. It significantly reduced intracellular ROS levels, protected against morphological changes, and prevented nuclear condensation. Furthermore, quercetin preserved the mitochondrial membrane potential, thereby inhibiting Cu-induced apoptosis. In conclusion, this study highlights the promising role of quercetin as a potential therapeutic agent in mitigating Cu-mediated neurotoxicity. By elucidating the diverse molecular signaling pathways involved, the research paves the way for further development of effective strategies to combat neurodegenerative diseases (Chakraborty et al., 2022).

#### 2.3.4 Genistein and daidzein

Park et al. (2010) explored the neuroprotective potential of two isoflavones, daidzein and genistein, against homocysteine-induced neurotoxicity in SH-SY5Y human neuroblastoma cells. Their study revealed promising mechanisms by which these isoflavones exert their protective effects. The treatment significantly suppressed homocysteine-mediated ER stress, evidenced by reduced expression of key ER stress markers: immunoglobin heavy chain-binding protein mRNA, spliced X-box-protein-1 mRNA, and phosphorylated eukaryotic translation initiation factor 2a (eIF2α) protein. This suggests that isoflavones directly target the ER stress pathway, potentially mitigating its detrimental effects on neuronal health. Furthermore, the study demonstrated a specific effect on tau protein hyperphosphorylation, a hallmark of neurodegenerative diseases like Alzheimer’s disease. Isoflavone treatment significantly reduced tau hyperphosphorylation through two key mechanisms: inactivation of glycogen synthase kinase 3β (GSK-3β) and activation of protein serine/threonine phosphatase 2A (PP2A). These findings highlight the potential of isoflavones to modulate tau phosphorylation pathways, a promising avenue for therapeutic intervention in neurodegenerative disorders.

#### 2.3.5 Isoflavones

Park et al. (2009) explored the neuroprotective potential of isoflavones in human neuroblastoma cells, focusing on their effects on apoptosis and tau phosphorylation. Their study revealed that isoflavones counteract ER stress, as evidenced by reduced expression of key markers like spliced X-box binding protein-1 mRNAs, immunoglobulin binding protein mRNA, and CHOP. Notably, isoflavones specifically inactivated glycogen synthase kinase 3β (GSK3β), a kinase implicated in tau hyperphosphorylation, leading to reduced tau hyperphosphorylation. These findings suggest that isoflavones may protect neurons by modulating both ER stress pathways and tau phosphorylation, potentially offering therapeutic benefits in neurodegenerative diseases. In contrast, Martin et al. (2013) reported a distinct mechanism for two flavonoids, honokiol (HNK) and EGCG, focusing on their interaction with GRP78, a key ER chaperone. While both flavonoids bind to unfolded GRP78 conformations, HNK exhibited a stronger affinity, leading to ER stress and cell death in various neuroectodermal tumor cell lines. Interestingly, EGCG proved less effective in inducing these effects. This study highlights the potential for GRP78-targeted compounds in antitumor strategies, but emphasizes the need for careful selection based on their specific interaction profiles and biological outcomes.

#### 2.3.6 Wogonin

Ge et al. (2015) delved into the anti-cancer potential of wogonin, a flavonoid extracted from the Chinese herb Scutellaria baicalensis, against malignant neuroblastoma cells. Their study explored the molecular mechanisms underlying wogonin-induced apoptosis, focusing on the IRE1α-dependent pathway. Treatment with varying doses of wogonin (0–150 μM) revealed that 75 μM for 48 h significantly promoted apoptosis in SK-N-BE2 and IMR-32 neuroblastoma cell lines. This was accompanied by the release of cytochrome c, a key step in the apoptotic pathway, and increased expression of ER stress-related proteins, indicating wogonin’s ability to trigger both mitochondrial dysfunction and ER stress in these cancer cells. Furthermore, the study specifically investigated the role of the IRE1α pathway, a stress response pathway known to be involved in apoptosis. The results strongly suggest that wogonin induces apoptosis in these cells through the modulation of this pathway.

#### 2.3.7 Juniper berry extract (*Juniperus communis* L.)

Lantto et al. (2016) explored the anti-tumor potential of juniper berry extract (*Juniperus communis* L.) against human neuroblastoma SH-SY5Y cells, focusing on its ability to induce p53-mediated apoptosis. P53, a well-known tumor suppressor protein often mutated in neurodegenerative diseases (Talebi et al., 2021), plays a crucial role in programmed cell death. The study revealed that juniper berry extract activated cellular relocalization and functionalization of p53, leading to DNA fragmentation and apoptotic cell death in neuroblastoma cells. Further analysis of gene expression changes identified 21 genes significantly overexpressed in treated cells compared to controls. These genes were associated with diverse cellular processes including protein synthesis, stress response, cell survival, and cell death. Notably, the upregulation of Heat Shock Protein Family A Member 5 (HSPA5), a known ER stress sensor, along with other ER stress-related genes like Calmodulin 2 (CALM2) and YKT6 V-SNARE Homolog (YKT6), suggested a link between ER stress response and juniper berry extract-induced apoptosis. In conclusion, this study provides evidence that juniper berry extract triggers p53-dependent apoptosis in neuroblastoma cells through multiple mechanisms, potentially involving ER stress pathways. Further research is warranted to elucidate the specific bioactive compounds responsible for these effects and explore the potential of juniper berry extract as a therapeutic agent in neuroblastoma treatment.

#### 2.3.8 Luteolin

Choi et al. (2011) unveiled the anti-tumor potential of luteolin, a dietary flavonoid, against Neuro-2a mouse neuroblastoma cells, elucidating its mechanism of action. This study demonstrates that luteolin triggers apoptosis in these cancer cells through a multi-pronged approach involving both ER stress and mitochondrial dysfunction. Luteolin treatment activated caspases, key executioners of programmed cell death, including caspase-12, -9, and-3, while regulating Bcl-2 family proteins involved in the intrinsic apoptotic pathway. Additionally, the study revealed ER stress induction evidenced by increased expression and activation of GRP 94, GRP 78, and CHOP proteins, cleavage of ATF6α, and phosphorylation of eIF2α. Interestingly, CHOP knockdown or treatment with the ER stress inhibitor, 4-phenyl butyric acid, mitigated luteolin-induced cell death, highlighting the crucial role of ER stress in this process. Furthermore, the study implicated mitochondrial dysfunction in luteolin’s cytotoxic effects. Luteolin rapidly reduced mitochondrial membrane potential, an effect countered by the antioxidant N-acetylcysteine. Remarkably, this antioxidant also reduced CHOP and GRP78 expression, suggesting a potential crosstalk between ER stress and mitochondrial dysfunction induced by luteolin. Additionally, luteolin activated p38, JNK, and ERK MAPKs, signaling pathways known to regulate mitochondrial function. Moreover, it triggered rapid mitochondrial translocation of Bax and cytochrome c release, key steps in the intrinsic apoptotic pathway, potentially via these MAPK pathways. In conclusion, this study paints a comprehensive picture of luteolin’s anti-tumor activity in neuroblastoma cells, involving ER stress induction, mitochondrial dysfunction, and modulation of signaling pathways. These findings pave the way for further exploration of luteolin’s potential as a therapeutic agent in neuroblastoma treatment.

#### 2.3.9 Genistein

Genistein (GST), an isoflavonoid derived from soybeans, exhibits diverse anticancer properties, including modulation of cell signaling pathways, cell cycle arrest, and apoptosis induction (Sarkar and Li, 2004). Additionally, fenretinide (4-HPR), a synthetic retinoid, has demonstrated efficacy in triggering apoptosis via ER stress and mitochondrial damage (Yeh et al., 2007). Recognizing these individual potentials, Karmakar et al. (Karmakar et al., 2011) investigated the synergistic potential of GST and 4-HPR in inducing apoptosis within human malignant neuroblastoma cells. Utilizing both *in vitro* models and *in vivo* xenograft models in nude mice, their study provided compelling evidence that the combined treatment potently activates multiple apoptotic pathways, culminating in significantly enhanced cell death compared to monotherapy. Mechanistically, this synergistic effect hinges upon the coordinated targeting of both ER stress and mitochondrial apoptotic pathways. This attack leads to the activation of pro-apoptotic proteins like Bax while simultaneously suppressing anti-apoptotic factors such as Bcl-2, ultimately tilting the balance toward cellular demise. Notably, down-regulation of the cell cycle regulator Id2 promotes differentiation, further hindering uncontrolled proliferation. Additionally, the combined treatment facilitates apoptosis execution by inhibiting members of the BIRC family, proteins renowned for impeding programmed cell death. Furthermore, activation of both caspase-3 and caspase-4, key executioners of apoptosis, ensures efficient cellular dismantling.

### 2.4 Fibromyalgia (FM)

Fibromyalgia, a chronic multifactorial syndrome affecting approximately 2% of the general population, predominantly females (90%), is characterized by widespread musculoskeletal pain, sleep disturbances, and fatigue (Bellato et al., 2012). While the exact cause remains elusive, various factors likely contribute to its development, including genetic predisposition, immunological abnormalities, stress infections, and hormonal fluctuations (Wolfe, 1994). Beyond these individual factors, the pathophysiology of fibromyalgia involves a complex interplay between genetics, aberrant neuroendocrine and autonomic nervous system activity, and environmental triggers such as stress exposure. Notably, these elements frequently overlap with co-occurring conditions like major depressive disorder (MDD), irritable bowel syndrome (IBS), and temporomandibular disorders (TMD) (Bradley, 2009).

#### 2.4.1 Fisetin

Fisetin (7,3′,4′- flavon-3-ol) is a flavonoid with antioxidant and anti-inflammatory qualities. In a reserpine-induced model of fibromyalgia, Ghoneim et al. (2021) explored the possible impacts of Fisetin. When the carotid artery and cerebral cortex of subjects within the control group were inspected, a high level of autophagy markers, light chain 3 (LC-3) and Beclin-1 (BECN-1), and apoptosis was found within the carotids as well as endothelial, vascular and cerebral cortex evidenced by a significant rise in caspase-3 expression and reduced Bcl-2 mRNA gene expression. The cerebral cortex was the same except ER stress-induced changes due to increased CHOP gene expression. Fibromyalgia-associated oxidative stress may be the reason for excessive apoptosis. An imbalance between antioxidant and oxidant generation might cause fibromyalgia-related disorders, reversible through Fisetin administration.

### 2.5 Spinal cord injuries (SCI)

#### 2.5.1 Wogonin

ER stress is critical in the pathogenesis of SCI (Ohri et al., 2011). In a study conducted by Chen et al. (2015) investigated the neuroprotective potential of wogonin against ER stress-induced damage in rat dorsal root ganglion (DRG) neurons. Pretreatment with wogonin significantly reduced markers of oxidative stress (MDA, SOD), ER stress (ATF4, p-PERK, p-eIF2α), and apoptosis (Bcl-2/Bax ratio, CHOP, GRP78) in tunicamycin (TUN)-induced DRG neurons. Notably, the number of TUNEL-positive neurons, indicating DNA fragmentation and apoptosis, was also significantly decreased. These findings strongly suggest that wogonin can effectively protect DRG neurons from ER stress-mediated damage. Building upon this work, Xu et al. (2015) investigated the mechanism of the wogonin neuroprotective effect on the rat DRG neurons. Wogonin preserves cell viability and prevents the apoptosis of TUN-induced DRG neurons. It also upregulated the level of GSH, inhibits the release of LDH, and prevents the expressions of ER stress markers, including glucose-regulated protein, glucose-regulated protein, CHOP, active caspase3, and active caspase12, in TUN-induced DRG Neurons. This study indicates that wogonin protects DRG neurons against TUN-induced ER stress.

### 2.6 Polyglutamine (poly Q) diseases

Polyglutamine (PolyQ) diseases are a group of nine inherited, autosomal dominant neurodegenerative disorders caused by the expansion of CAG trinucleotide repeats in specific genes. These genetic alterations result in the production of proteins containing abnormally long stretches of glutamine residues (polyglutamine tracts) (Cohen-Carmon and Meshorer, 2012; Bunting et al., 2022). Notably, PolyQ diseases are classified as late-onset disorders, with symptoms typically manifesting in adulthood and progressively worsening over a period of 10 to 30 years (Paulson et al., 2000).

#### 2.6.1 Naringenin

In a study by Yamagishi et al. (2012), naringenin, a flavonoid found in citrus fruits, emerged as a potential therapeutic candidate for polyglutamine (polyQ) diseases. The study found that GRP78, a key ER chaperone protein responsible for protein folding and quality control, was significantly reduced in cells expressing a pathological-length polyQ tract, suggesting its depletion contributes to disease progression. Interestingly, the researchers observed an inverse relationship between GRP78 levels and polyQ protein aggregation, highlighting the importance of maintaining proper ER function in these diseases. Aiming to identify compounds that could restore GRP78 expression, Yamamoto et al. screened various herbal extracts. Notably, naringenin potently induced GRP78 expression in multiple cell lines, indicating its potential to alleviate ER stress in polyQ disease models. Furthermore, the study demonstrated that naringenin suppressed both apoptosis (programmed cell death) and protein aggregation induced by the mutant polyQ protein. These findings collectively suggest that naringenin holds promise as a therapeutic agent for polyQ diseases by acting as a potent inducer of GRP78, thereby mitigating ER stress, suppressing apoptosis, and preventing protein aggregation.

## 3 Alzheimer’s disease (AD)

Alzheimer’s disease (AD), affecting over 25 million people globally and accounting for more than 75% of all dementia cases, poses a significant healthcare challenge (Ullah, 2015; Qiu et al., 2022). This progressive neurodegenerative disorder arises from a complex interplay of both genetic and non-genetic factors. While genetics are crucial in both late-onset and early-onset forms of AD, numerous non-genetic contributors play a key role in disease initiation. These include pre-existing conditions like diabetes, cancer, depression, and cardiovascular disease, as well as occupational exposures, smoking habits, and lifestyle factors like physical and cognitive activity (Jiang et al., 2013). The hallmarks of AD pathogenesis include the extracellular accumulation of amyloid-β plaques, the intracellular formation of neurofibrillary tangles, neuroinflammatory cascades, and oxidative damage to neurons. However, abnormal increases in amyloid-β peptide production, the main component of amyloid plaques, are widely considered the central driver of the disease process (Imbimbo et al., 2005).

### 3.1 Quercetin

**Figure 2:**
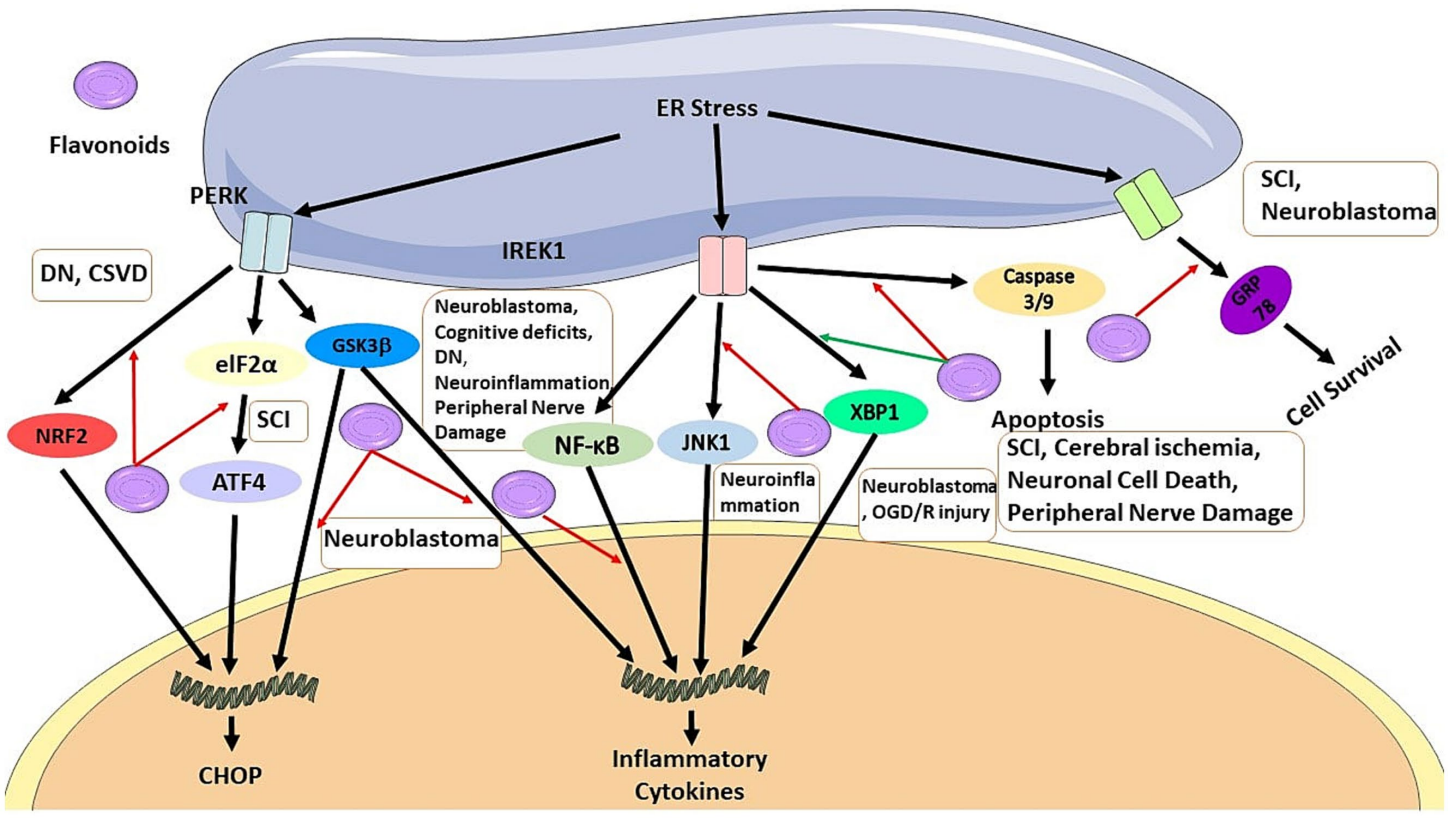
Flavonoids can inhibit CHOP pathway by blocking NRF2 signaling pathway in DN and CSVD, by blocking elF2a/ATF4 pathway in SCI, and GSK3b in neuroblastoma. It also can inhibit production of inflammatory cytokines by blocking GSK3b in neuroblastoma, and by blocking NF-KB pathway in neuroblastoma, cognitive deficts, DN, neuroinflammation, and peripheral nerve damage, and also by activation of XBP1 pathway in neuroblastoma and OGD/R injury, it can regulate ER stress. Flavonoids has anti-apoptosis effects by inhibiting caspase 3/9 production in SCI, cerebral ischemia, neuronal cell death, peripheral nerve damage. It also can result in cell survival by regulating GRP78.

Hayakawa et al. (2015) investigated the potential of quercetin, a plant flavonoid, to improve memory in an AD mouse model. AD, characterized by memory decline and progressive neurodegeneration, involves complex mechanisms including impaired protein degradation (autophagy) and ER stress. Prior studies linked these factors to increased production of amyloid-β peptides, a hallmark of AD pathology. They focused on eIF2α phosphorylation, a key stress response pathway implicated in AD. Chronic activation of kinases in this pathway leads to persistent eIF2α phosphorylation, hindering general protein translation while selectively allowing the translation of certain stress-related proteins like activating transcription factor 4 (ATF4). ATF4, in turn, can further worsen ER stress and amyloid-β production. The study revealed that quercetin treatment reduced eIF2α phosphorylation and ATF4 expression in the brains of AD mice. This effect was mediated by growth arrest and DNA damage-inducible gene 34 (GADD34) induction, a protein known to inhibit eIF2α phosphorylation. Consequently, memory function was significantly improved in quercetin-treated mice compared to controls, suggesting a potential therapeutic benefit at the early stages of AD (Figure 2). Liu et al. (2020) explored a promising approach for delivering quercetin to the brain using focused ultrasound and microbubbles. This study aimed to bypass the BBB by encapsulating quercetin-modified sulfur nanoparticles (QcSNPs) within microbubbles and using focused ultrasound to enhance their delivery to the brain. Microbubbles act like tiny balloons, temporarily opening the BBB upon exposure to ultrasound waves, allowing QcSNPs to reach the brain parenchyma (tissue). Compared to directly injecting QcSNPs, this method led to greater accumulation of QcSNPs in the brain, as demonstrated by the rapid increase in QcSNP concentration following focused ultrasound treatment. This enhanced delivery resulted in more effective reduction of neural apoptosis (cell death) and mitigation of ER stress-related problems like oxidative stress, calcium imbalance, and inflammation. These positive effects ultimately translated to improved memory and learning abilities in AD mice as assessed by the Morris water maze test. Overall, this study highlights the potential of focused ultrasound-mediated microbubble delivery as a promising strategy for effectively delivering therapeutic agents like quercetin to the brain for AD treatment (Supplementary Table S1).

Another research team (Panche et al., 2016) investigated its potential to regulate the integrated stress response (ISR) and improve memory. This study specifically focused on ER stress signaling, a key component of the ISR, and its role in AD pathogenesis. The researchers reviewed existing evidence suggesting that excessive ER stress contributes to cognitive impairments in AD mice. Importantly, their findings demonstrated that quercetin can effectively inhibit ER stress signaling and alleviate memory deficits in these AD models. Furthermore, the study explored the potential of quercetin to promote neurogenesis, the birth of new neurons, and upregulate brain-derived neurotrophic factor (BDNF), a protein crucial for neuronal survival and growth. These combined effects suggest that quercetin may help slow down the progression of AD, particularly in the early stages. Beyond AD, the study highlighted the broader potential of ISR inhibitors, like quercetin, in preventing cognitive deficits associated with traumatic brain injury (TBI) and neurodegeneration in prion diseases, opening avenues for further research in these areas.

Further studies are needed to identify the target molecules implicated in ESR signaling in neuronal disease and possible therapeutic interventions. Regitz et al. (2014) investigate the effect of dietary polyphenols in preventing AD. Notably, their study found that increasing quercetin dosage led to a dose-dependent reduction in protein aggregates within the research sample. Interestingly, silencing key components of the unfolded protein response (UPR) using RNA interference (RNAi) did not completely abolish protein aggregation in the nematode model of AD, suggesting limitations to this approach. However, the study revealed that the proteostasis network, encompassing the UPR, plays a role in Aβ1-42 aggregation and associated paralysis symptoms. Furthermore, quercetin demonstrated its ability to activate both macroautophagy and proteasomal degradation pathways, two key cellular mechanisms for protein clearance. This enhanced protein clearance capacity allowed quercetin to prevent Aβ1-42 aggregation and paralysis in the model organism, suggesting its potential to potentially prevent AD onset. Ohta et al. (2011) investigate the ER stress enhances c-secretase activity. They understand that ER stress decreases presenilin-1 expression by ATF4, which improves amyloid-b secretion by c-secretase and can cause the pathogenesis of diabetes and obesity, risk factors for AD. Also, they understand that quercetin can decrease this stress by modifying UPR signaling.

### 3.2 Baicalein

Exploring the neuroprotective potential of flavonoids in AD, Obulesu and Lakshmi (2014) examined the capacity of baicalein, a flavonoid isolated from Scutellaria baicalensis Georgi, to mitigate apoptosis in a mouse hippocampal neuronal HT22 cell model. Employing thapsigargin (TG) and brefeldin A (BFA) as inducers of ER stress and subsequent apoptosis, the researchers assessed baicalein’s protective effects. Their findings revealed that baicalein significantly prevented apoptosis triggered by both TG and BFA. Mechanistically, baicalein demonstrably activated and cleaved caspase-12 and-3, known executioners of apoptosis, suggesting its direct engagement in the apoptotic cascade. Additionally, baicalein downregulated the expression of ER stress-associated protein CHOP, indicating its potential to alleviate ER stress and its contribution to apoptosis.

### 3.3 Luteolin

Recent studies showed luteolin as a promising warrior against AD (Goyal et al., 2024). In a study by Kou et al. (2022), Luteolin, a naturally occurring flavonoid, demonstrated promising neuroprotective and memory-enhancing effects in a mouse model of AD. Treatment with Luteolin for three weeks resulted in dose-dependent improvement in spatial learning and memory in AD mice. This cognitive enhancement was linked to attenuated neuroinflammation and astrocyte activation observed in their brains. Mechanistically, Luteolin reduced the expression of ER stress markers in brain tissues, suggesting its ability to alleviate cellular stress and promote neuronal health. Further investigations in rat C6 glioma cells revealed that Luteolin suppressed the uncontrolled release of inflammatory cytokines and lipopolysaccharide (LPS)-induced cell proliferation. Additionally, it demonstrated a dose-dependent increase in GRP78, a key ER chaperone protein, indicating its potential to restore ER homeostasis. These findings suggest that Luteolin’s effectiveness in improving learning and memory deficits is mediated by its ability to reduce ER stress in astrocytes, thereby preventing the cascade of neuroinflammation and neuronal damage hallmarking AD.

## 4 Parkinson’s disease (PD)

Parkinson’s disease (PD) ranks second among neurodegenerative disorders in prevalence, characterized by the progressive destruction of dopaminergic neurons in the substantia nigra pars compacta (Meara and Hobson, 2018). This leads to the three cardinal motor symptoms: tremor, bradykinesia (slowness of movement), and rigidity. Affecting approximately 10–50 individuals per 100,000, PD prevalence escalates with age and exhibits a slight male predominance (Elbaz et al., 2016).

While the exact cause of PD remains elusive, a complex interplay of genetic and environmental factors is evident (Wirdefeldt et al., 2011). Pathologically, the disease is hallmarked by the presence of round, eosinophilic, intracytoplasmic inclusions called Lewy bodies (LBs) and dystrophic neurites (Lewy neurites) within surviving neurons (Forno, 1981). Notably, PD primarily presents as a sporadic disease, but dedicated research efforts in genetics, epidemiology, neuropathology, and innovative experimental models have illuminated crucial insights into its development and progression (Moore et al., 2005).

### 4.1 Quercetin

El-Horany et al. (2016) investigated the potential of quercetin to mitigate PD progression in a rotenone-induced rat model. This study explored quercetin’s ability to improve both behavioral and neurochemical deficits observed in these PD rats. Their findings revealed that quercetin treatment induced autophagy, a cellular recycling process, and consequently reduced behavioral impairments associated with rotenone exposure. Additionally, quercetin attenuated ER stress-mediated apoptosis and oxidative stress, suggesting its ability to protect neurons from multiple damaging pathways in PD. These results indicate that quercetin could act as an autophagy enhancer in PD models, ultimately modifying the cellular environment to prevent neuronal death. Park and Chun (2016) further explored the neuroprotective effects of quercetin in a different PD model, examining its potential to combat apoptosis and ER stress induced by dieldrin, a pesticide linked to PD development. Their study focused on dopaminergic neuronal cells, which are particularly vulnerable in PD. The results demonstrated that pre-treatment with quercetin significantly suppressed dieldrin-induced apoptotic markers like nuclear condensation, DNA fragmentation, and caspase-3/7 activation in these dopaminergic cells. Notably, quercetin also prevented changes in ER stress markers caused by dieldrin exposure. These findings suggest that quercetin may play a role in preventing dieldrin-induced neuronal apoptosis, offering a potential avenue for PD prevention (Figure 2).

### 4.2 Morin

Morin, a naturally occurring bioflavonoid, exhibits promising neuroprotective potential in PD models, according to studies by Lee et al. (2016) and Zhang et al. (2010). Lee et al. (2016) investigated the effects of morin in a chemically-induced PD mouse model. Their study revealed that morin significantly improved motor deficits, prevented dopaminergic neuron loss, and reduced astroglial activation, a marker of inflammatory response. Morin exerted its neuroprotective effects by modulating mitochondrial dysfunction and reducing ROS formation, both major contributors to neuronal damage in PD. Additionally, morin dampened inflammation and reactive astrocytosis, further protecting neurons from detrimental processes. These findings suggest Morin’s potential as a multifaceted therapeutic agent for PD treatment and prevention. Zhang et al. (2010) explored the efficacy of morin in another PD mouse model. This study demonstrated that morin treatment alleviated Parkinsonian symptoms, evidenced by improved behavioral performance and reduced dopaminergic neuron loss. These findings further bolster the potential of morin as a neuroprotective agent in PD. Overall, these studies highlight the promising potential of morin in mitigating key pathological features of PD, including oxidative stress, mitochondrial dysfunction, and neuroinflammation (Supplementary Table S1).

### 4.3 Rutin

Enogieru et al. (2019) investigated the neuroprotective effects of rutin, a plant flavonoid PD cellular model. They employed MPP + -treated SH-SY5Y neuroblastoma cells, which mimic key features of PD, and examined the impact of rutin on various cellular stress responses. Their findings revealed that MPP+ treatment alone increased intracellular calcium levels (Ca2+), triggered ER stress, and impaired mitochondrial function, including bioenergetic status and membrane potential. Importantly, the study provides the first evidence that rutin can alleviate these detrimental effects in this PD model. Mechanistically, the researchers discovered that rutin facilitates the maintenance of Ca2+ homeostasis, inhibits ER stress, and protects mitochondria in MPP + -treated cells (Kaufman, 1999; Ghribi et al., 2003). They focused on BiP, a key chaperone protein activated during ER stress (Lowenstein et al., 1991; Yu et al., 1999), which plays a crucial role in protecting neurons from damage and ensuring proper protein folding (Ghribi et al., 2003). They hypothesized that rutin-induced upregulation of BiP might be central to its neuroprotective effects. Their results demonstrated that rutin significantly increased BiP expression, suggesting that it activates the unfolded protein response (UPR), a cellular pathway that alleviates ER stress and promotes proper protein folding. This finding implies that rutin’s protective activity in this PD model likely stems from its modulation of the UPR and subsequent reduction of ER stress (Enogieru et al., 2019).

## 5 Cerebral ischemia

The middle cerebral artery, other large cerebral arteries, *in situ* thrombosis related to an atherosclerotic plaque, or embolism from a proximal area are all factors in the pathophysiology of stroke, including bleeding into the brain tissue from ruptured brain aneurysms (Kim and Kim, 2014).

### 5.1 Morin

Exploring morin’s potential in cerebral ischemia treatment, Chen et al. (2017) investigated its effects in a male rat model of focal cerebral ischemia, a condition mimicking stroke. They treated the rats with morin for seven days and observed reduced neurological deficits. This improvement was linked to increased levels of antioxidant enzymes, suggesting morin’s ability to boost cellular defenses against harmful ROS generated during stroke. Furthermore, the study revealed increased expression of anti-apoptotic proteins like Bcl-2, indicating morin’s capacity to protect cells from programmed cell death (apoptosis). Conversely, pro-apoptotic proteins like Bax and caspase-3, key regulators of mitochondrial cell death pathways, were downregulated, further supporting morin’s anti-apoptotic effects.

## 6 Diabetic neuropathy (DN)

DN is the most important complication of DM, which has an accuracy of 50% in all diabetic patients. It’s the main reason for 50–75% of no traumatic amputations (Vinik et al., 2013). Hyperglycemia is the most common cause of DN. The main mechanism of DN is still unclear. However, many probable etiologies for DM, including elevated superoxide-induced free radicals, disrupted metabolism of lipids, amino acids, and insufficiency of vessels, have been considered (Feldman et al., 1997).

### 6.1 Morin

Bachewal et al. (2018) explored the potential of morin, a natural flavonoid with antioxidant and anti-inflammatory properties, in alleviating DN symptoms. Utilizing an experimental DN model, their study demonstrated that morin administration effectively reduced pain hypersensitivity (hyperalgesia and allodynia) and improved nerve conduction. The research suggests that morin’s beneficial effects stem from its ability to counteract the detrimental effects of high glucose on mitochondrial ROS production, a key contributor to nerve damage in DN. Morin inhibited ROS-mediated activation of IKK, a critical molecule in the NF-κB pathway linked to neuroinflammation. Furthermore, it enhanced Nrf2-mediated antioxidant defenses, boosting the body’s natural defenses against ROS. These findings, coupled with morin’s overall antioxidant and anti-inflammatory properties, highlight its potential as a therapeutic agent for DN treatment. Notably, activation of the NF-κB pathway in DN leads to increased levels of COX2 and thromboxane, promoting vasoconstriction and myelin damage via inflammation. By mitigating these processes, morin may offer neuroprotective benefits. Additionally, by restoring Nrf2 activity, which is often reduced in DN, morin can further enhance cellular antioxidant defenses (Pop-Busui et al., 2002; Wakabayashi et al., 2010).

## 7 Stroke

The definition of stroke is a set of rapidly developing signs and symptoms of focal loss of function in the brain that can progress to a global loss of function (patients with subarachnoid hemorrhage or those with deep coma) (Warlow, 1998). Hypertension, diabetes, high blood homocysteine levels, and genetic factors are the main risk factors for stroke (Lo et al., 2003). The incidence of stroke is 2–3/1000 people, and the prevalence is five in 1000 people, and its causes of death of 4.5 million people per year (Wolfe, 2000).

### 7.1 Liquiritin

Stroke is known as one of the leading causes of death worldwide. Today, thrombolytic treatment is one of the treatment strategies, but due to its tight time window, only a small number of people can benefit from it. This necessitates the exploration of novel therapeutic strategies. Liquidation (LQ) is a flavonoid that derived from the plant Glycyrrhiza uralensis Fisch. The article by Li et al. (2021) investigated the action of LQ on brain microvascular endothelial cells (BMECs) and presented a new therapeutic strategy for treating stroke. As a result, LQ decreases apoptosis and potential damage to the mitochondrial membrane. Meantime, it can also reduce ROS production. These functions of LQ can prevent ER stress and maintain BBB integrity.

## 8 Neuronal cell death

Pathological protein synthesis and, in many circumstances, the creation of high-order aggregates are frequently associated with neurodegenerative disease traits (Soto, 2003; Blokhuis et al., 2013; Michel et al., 2016). These factors frequently cause neurons to be under stress, which in turn causes cytotoxic events such as an increase in excitotoxicity, ROS, synaptic dysfunction, ER stress, impaired protein degradation systems, DNA damage, cell cycle re-entry, inflammation, and mitochondrial dysfunction (Seward et al., 2013).

### 8.1 Quercetin

Chakraborty et al. (2022) investigated the neuroprotective potential of quercetin against copper-induced cell death in the SH-SY5Y neuroblastoma cell line. This model exhibits neurotoxic effects and balanced apoptotic cell death, mimicking key features of PD. The study revealed that quercetin displayed superior cell protection compared to other polyphenols. It effectively shielded SH-SY5Y cells from copper-induced cellular death, morphological abnormalities, and oxidative stress. Notably, quercetin modulated the expression of α-synuclein protein and chaperone-mediated autophagy, key players in PD pathogenesis. This modulation, crucially, prevented neuronal apoptosis by mitigating ER stress, a major contributor to neurodegeneration. Furthermore, quercetin treatment facilitated maintenance of cellular homeostasis in neurons. These findings suggest its potential therapeutic application in PD-like disorders by reducing apoptotic features and restoring mitochondrial health.

### 8.2 Qenistein

Linford and Dorsa (2002) investigated the potential of genistein, a soy isoflavone, to reduce neuronal cell death through an estrogen receptor-dependent mechanism. They employed a model of apoptosis toxicity in primary cortical neurons, induced by thapsigargin, an inhibitor of the ER calcium ATPase. Their study aimed to compare the anti-apoptotic effects of genistein and 17β-estradiol, the endogenous estrogen. Interestingly, both genistein and 17β-estradiol treatment significantly reduced the number of apoptotic neurons, as well as the number of neurons expressing active caspase-3, a key executioner of programmed cell death. Notably, this protective effect was completely abolished by co-administration of ICI 182,780, a specific inhibitor of estrogen receptors. These findings suggest that genistein’s neuroprotective ability against apoptosis in this model appears to be mediated through estrogen receptor signaling, similar to 17β-estradiol.

## 9 Peripheral nerve damage

### 9.1 Quercetin

Yardim et al. (2020) explored the neuroprotective potential of quercetin against vincristine-induced peripheral nerve damage in rats. Vincristine, a chemotherapeutic agent, is known to cause neurotoxicity by decreasing levels of the antioxidant protein Nrf2 and its downstream targets HO-1 and NQO1. This leads to oxidative stress, DNA damage, inflammation, endoplasmic reticulum (ER) stress, and apoptosis in neuronal cells. The study demonstrated that quercetin treatment effectively counteracted these detrimental effects of vincristine. It reduced oxidative stress and DNA damage, as evidenced by decreased levels of 8-OHdG. Additionally, quercetin mitigated inflammation and ER stress, indicated by reduced levels of GFAP, NF-κB, PERK, IRE1, ATF-6, and GRP78. Furthermore, it prevented apoptosis by decreasing caspase-3 and Bax expression and increasing Bcl-2 expression. These findings suggest that quercetin exerts its protective effects through activation of antioxidant, anti-inflammatory, and anti-apoptotic mechanisms. Notably, the study also observed increased Akt activation following quercetin treatment, suggesting a potential involvement of this pro-survival pathway.

## 10 Cerebral small vessel disease (CSVD)

CSVD refers to the wide spectrum of cerebrovascular disease with different etiologies and pathogenesis that have a large impact on venules, capillaries, and arterioles which supply the white matter and deep parts of the brain (Chojdak-Łukasiewicz et al., 2021). Senile and elevated blood pressure related to small vessel disease and amyloid deposition angiopathy are three most common types of CSVD (Pantoni, 2010).

### 10.1 Quercetin

Li et al. (2021) investigated the potential protective effect of quercetin on endothelial cell damage and prepared a basic theory for further application in the clinic. They showed that quercetin promotes the livability, angiogenesis, and migratory capacity of human brain microvascular endothelial cells (HBMECs) and prevents apoptosis. They reported that quercetin preserves HBMECs from hypoxia and reoxygenation (H/R), which includes cell multiplication, cell migration, angiogenesis promotion, mitochondrial membrane potential injury reduction, cell apoptosis inhibition in terms of its antioxidation, and prevention of ER stress. Concurrent quercetin can enhance the BBB connexin level and retain BBB integrity by increasing the claudin-5 and ZO-1 protein rates. Most importantly, H/R could result in ER stress and increase ER stress-related proteins. Furthermore, quercetin could enhance increased Keap1/Nrf2 levels and increase the antioxidant capacity of HBMECs, significantly down-regulate AFT6/GRP78 protein, interdict ER stress, and protect HBMECs from H/R damage.

## 11 Liver disease

### 11.1 Fisetin

Dai et al. (2022) investigated the potential of fisetin, a naturally occurring flavonoid, in alleviating non-alcoholic fatty liver disease (NAFLD) symptoms. Their study focused on its ability to protect hepatocytes, liver cells, from damage caused by palmitate (PA), a fatty acid implicated in NAFLD development. The research revealed that fisetin significantly reduced ROS generation in both human and mouse hepatocytes exposed to PA. This suggests its antioxidant potential in mitigating oxidative stress, a key contributor to NAFLD progression. Additionally, fisetin treatment effectively prevented PA-induced mitochondrial dysfunction and impairment, highlighting its ability to protect these energy-producing organelles. Furthermore, the study observed that fisetin significantly suppressed cytochrome-c release and mitochondrial apoptosis in PA-treated hepatocytes. This indicates its capacity to prevent programmed cell death triggered by cellular stress. Notably, fisetin treatment also led to a marked reduction in PA-induced lipid deposition within hepatocytes, showcasing its potential in addressing fat accumulation, a hallmark of NAFLD. The researchers found that deleting Nrf2, a key regulator of cellular antioxidant responses, abolished fisetin’s protective effects against apoptosis, inflammation, and lipid accumulation.

### 11.2 Bavachin

Yang et al. (2021) explored the potential role of Sestrin2, a stress-responsive protein, in mitigating liver damage caused by bavachin, a naturally occurring flavonoid with hepatotoxic (liver-damaging) properties. Their study employed two separate models: mice treated with bavachin and cultured HepG2 liver cells exposed to bavachin. In mice, the study found that bavachin triggered ER stress, a cellular imbalance associated with liver injury. Interestingly, increased levels of Sestrin2 protein were observed alongside this ER stress. Subsequent experiments in HepG2 cells, using siRNA to silence Sestrin2, revealed that Sestrin2 depletion indeed worsened bavachin-induced ER stress. This suggests that Sestrin2 plays a protective role against bavachin’s harmful effects. The researchers propose that Sestrin2 exerts its protective action by modulating the AMPK/mTORC1 pathway, a key cell growth and energy regulation pathway. When Sestrin2 is depleted, mTORC1 activity remains high, leading to accumulation of misfolded proteins within the ER, further amplifying ER stress. Overall, this study suggests that Sestrin2 may act as a natural defense mechanism against bavachin-induced liver damage by regulating the AMPK/mTORC1 pathway and preventing ER stress overload.

### 11.3 Quercetin

Tang et al. (2012) investigated the potential of quercetin, a natural flavonoid, to protect against ethanol-induced liver damage and dyslipidemia, specifically focusing on its effects on mitochondrial oxidative stress. Their study demonstrated that prophylactic administration of quercetin significantly mitigated ethanol-induced mitochondrial damage in rats. This was evidenced by improved mitochondrial membrane potential, reduced permeability transition pore opening, and decreased levels of oxidative stress markers such as glutathione depletion, enzymatic inactivation of antioxidant enzymes (MnSOD and glutathione peroxidase), ROS generation, and lipid peroxidation within the mitochondria.

## 12 Miscellaneous

### 12.1 Baicalin

Lin et al. (2014) employed an *in vitro* model using the human renal proximal tubular epithelial cell line HK-2. They exposed these cells to H2O2, a stressor mimicking oxidative damage, with and without pre-treatment with Baicalin, a natural flavonoid known for its anti-inflammatory properties. The study observed that H2O2 treatment significantly reduced cell viability and increased apoptosis (programmed cell death) in HK-2 cells. Additionally, analysis of ROS and the glutathione ratio (GSH/GSSG) revealed a marked increase in oxidative stress within the cells exposed to H2O2. However, pre-treatment with Baicalin prior to H2O2 exposure exhibited a protective effect. It significantly improved cell viability and reduced apoptosis. Further investigation revealed that Baicalin’s protective mechanism involves inhibiting ER stress and activating the downstream Nrf2 signaling pathway, a key regulator of cellular antioxidant responses. These findings suggest that Baicalin may hold promise in protecting against renal damage caused by oxidative stress by mitigating ER stress and enhancing antioxidant defenses.

### 12.2 α-ZOL and β-ZOL

Ben Salem et al. (2016) investigated the cellular toxicity mechanisms of α-ZOL and β-ZOL in HCT116 cells and explored the potential protective role of quercetin. Their study revealed that both α-ZOL and β-ZOL treatment induced ER stress and activated the UPR, indicating cellular stress leading to potential damage. Combining α-ZOL or β-ZOL treatment with quercetin significantly reduced the detrimental effects observed in various toxicity markers. This suggests that quercetin possesses protective properties against the cellular toxicity caused by α-ZOL and β-ZOL.

## 13 Other neuroprotective properties of flavonoids

### 13.1 Rutin

Mostafa et al. (2019) investigated whether rutin hydrate (RH), a recognized antioxidant flavonoid and neuroprotective, can reduce cadmium chloride (CdCl2)-induced neurotoxicity using inhibiting ER stress. Induced activation of ER stress and increased oxidative stress were observed in brain homogenates from CdCl2-treated rats, as evidenced by increased protein and mRNA levels of X-box-binding protein 1 (XBP1), CHOP, GRP78, and ATF-6, as well as protein levels of p-JNK1/2, cleaved caspase-12, and p-elF2α. In the brains of both control and CdCl2-treated rats, RH increased glutathione (GSH) levels and activities of Plasma glutathione peroxidase (GSH-Px), and Superoxide dismutase (SOD) decreased Malondialdehyde (MDA) levels and inhibited mitochondrial permeability transition pore (mtPTP). Therefore, RH decreased all markers of ER stress, increased Bcl-2, decreased mitochondrial Bax translocation, and improved mitochondrial coupling in brain homogenates of CdCl2-treated rats. Overall, the findings support RH’s ability to inhibit ER stress in the brains of CdCl2-treated rats and its other neuroprotective mechanisms.

### 13.2 Fisetin

In a study by Yen et al. (2017), the effects of a flavonoid substance, Fisetin, on neuronal-like catecholaminergic PC12 cells were investigated. The cells were subjected to the cytotoxic substance tunicamycin, and then one group of cells was treated with low-dose Fisetin to investigate if it could prevent cytotoxicity in these neuronal-like cells. The results elucidated that low-dose Fisetin protects PC12 cells from tunicamycin-induced cell death through alleviating ER stress and unfolded protein response (UPR) and restoring expression of factors like Sirtuin 1 (SIRT1). Tunicamycin (TUN), an antibiotic synthesized by Streptomyces lysosuperificus, can also chemically induce ER stress, a state of dysfunction in the ER to the accumulation of misfolded proteins inside the lumen. These misfolded proteins activate UPR, resulting in higher expression of proteins that downstream relieve ER stress (Lai et al., 2007). However, if ER stress is prolonged, cell death and apoptosis follow (Rasheva and Domingos, 2009). Like many other flavonoids, Fisetin has antioxidant, anti-inflammatory, and neuroprotective effects properties. It should be considered that at higher doses, it might be cytotoxic to cancer cells. It might exert these actions through the upregulation of SIRT1, nuclear E2-related factor 2 (Nrf2), and MAPKs, leading to higher resistance against oxidative stress and improved cell survival (Pearson et al., 2001; Magesh et al., 2012; Hubbard and Sinclair, 2014).

### 13.3 Liquiritigenin

Ramalingam et al. reviewed the qualities of Radix Glycyrrhizae. Some active ingredients of this old herbal medicine are flavanone liquiritigenin and its precursor, isoliquiritigenin. Administrating the agents as mentioned above show positive results in Glioma, stroke, and AD, as well as Parkinson’s disease, and many of these effects, are attributed to their anti-ROSs scavenging properties (Ramalingam et al., 2018). Polyhydroxylated flavonoids, namely, rutin, F7, F32, F11, F12, and F2.

### 13.4 Baicalein

Choi et al. (2010) investigated the effects of baicalein on hippocampal neuron apoptosis in HT22 mice induced by thapsigargin and brefeldin A. They showed that baicalein could protect HT22 neuronal cells from ER stress-induced cell death by reducing CHOP induction besides mitochondrial damage and ROS accumulation.

### 13.5 Quercetin

Chen et al. (2020) examined the effects of quercetin on Ca2+ homeostasis in mouse brains. They demonstrated that quercetin increases Ca2+ levels in a concentration-dependent manner. Quercetin-triggered Ca2+ signaling consists of both internal Ca2+ release and extracellular Ca2+ influx, and most importantly, quercetin-induced Ca2+ release from the ER, and as a result, inositol IP3R inhibition by xestospongin C blocked quercetin-induced Ca2+ release. Their work demonstrated that quercetin disrupts internal Ca2+ storage and inhibits ATP-triggered responses. Their results showed that quercetin mobilized Ca2+ from the ER without emptying this organelle. The data blocking IP3R by xestospongin C could suppress quercetin-triggered Ca2+ release supports this. Changes in ER homeostasis, consisting of severe Ca2+ deficiency, are upstream events in the pathophysiology of several diseases.

On the one hand, the inadequate release of the activator Ca2+ can no longer maintain important cellular functions, and loss of luminal Ca2+ causes ER stress, an unfolded protein that can restore normal ER function or cause cell death, depending on the duration and severity of the stress, activates the response (Mekahli et al., 2011). Thus, pathological neuronal loss in mature CNS is irreversible in motor and cognitive function, from acute CNS injuries such as TBI and ischemic stroke to chronic neurodegenerative diseases such as PD and AD (Fricker et al., 2018).

### 13.6 Genistein

Park et al. (2016) investigated the effects of dietary genistein supplementation on the hippocampus and cortex of wild-type C57BL/6 (WT) and ApoE knockout (ApoE−/−) mice that were given a high-fat diet that lasted 24 weeks. They showed that in ApoE−/− mice, genistein supplementation reduced presenilin one mRNA and beta-secretase 1 level and beta-amyloid peptide (Aβ) protein levels, but not in WT mice. Although the absence of ApoE did not affect tau hyperphosphorylation, genistein supplementation in both WT and ApoE−/− mice reduced tau hyperphosphorylation. Also, they found that genistein alleviated the activity of glycogen synthase kinase 3β and c-Jun N-terminal kinase, which are engaged in tau hyperphosphorylation.

### 13.7 Proanthocyanidins

Liu et al. (2014) investigated the protective effects of Proanthocyanidins (PCs) against lead-induced inflammation in the rat brain and studied its potential mechanism of action. They demonstrated that in the brains of lead-exposed rats, PCs meaningfully reduced the levels of cyclooxygenase-2, tumor necrosis factor-a, and interleukin 1b. Furthermore, PCs significantly decreased phosphorylated tau levels and beta-amyloid in the brains of lead-treated rats, inhibiting ER stress. PCs also inhibited nuclear factor-jB nuclear translocation and decreased eukaryotic translation initiation factor-2, phosphorylation of protein kinase RNA-like ER kinase, inositol-requiring protein-1, p38, and c-Jun N-terminal kinase in the brains of lead-exposed rats.

## 14 Traumatic brain injury (TBI)

### 14.1 Naringenin

The study conducted by Deng et al. (2021) indicates that naringenin can attenuate ER stress and decreases apoptosis. The study’s results showed that continued use of naringenin improves neurological dysfunction and permeability of plasmalemma. The authors mentioned that this substance could be effective by pleiotropic effects on TBI, including attenuation of ER stress.

## 15 Conclusion

Based on evidence from numerous studies, flavonoids appear as promising therapeutic candidates for diverse neurological disorders, particularly due to their ability to modulate endoplasmic reticulum (ER) stress. This ubiquitous cellular stress response plays a central role in the pathogenesis of several neurodegenerative diseases. The reviewed studies demonstrate that various flavonoids, including kaempferol, quercetin, luteolin, vogonin, rutin, apigenin, and morin, exhibit neuroprotective effects by mitigating ER stress in different cellular models.

Quercetin stands out as the most extensively studied flavonoid, demonstrating its protective role in both neuronal and endothelial cells. It effectively reduces key ER stress markers like GRP78 and CHOP while influencing intracellular calcium signaling, indicating its multifaceted approach to cellular stress management.

Other flavonoids such as luteolin and apigenin exhibit promising antitumor activity against glioblastoma cells by inducing apoptosis and targeting ER stress pathways. This opens avenues for exploring their potential as therapeutic agents in cancer treatment.

Furthermore, findings suggest the neuroprotective potential of rutin and morin in Parkinson’s disease models, highlighting their broader applicability in neurodegenerative disorders.

While these studies provide compelling evidence for the therapeutic potential of flavonoids in neurological disorders, further research is crucial to elucidate their precise mechanisms of action, optimize their delivery methods, and assess their efficacy and safety *in vivo* models and clinical trials. Understanding the specific interactions between individual flavonoids and various disease processes will pave the way for personalized therapeutic strategies targeting ER stress and other key hallmarks of neurodegeneration.

## Author contributions

MA: Writing – original draft, Writing – review & editing. MP: Writing – review & editing. BA: Writing – original draft. MY: Writing – original draft. MN: Writing – original draft. FM: Writing – original draft. MA: Writing – original draft. IA: Writing – original draft. FB: Writing – original draft. AB: Writing – original draft. PG: Writing – original draft. SI: Writing – original draft. AAA: Writing – original draft. AS: Writing – original draft. NZ: Writing – original draft. DA: Writing – original draft. HG: Writing – original draft. AA: Writing – original draft.

## Figures and Tables

**Table 1:** 

4-HPR	N-(4-hydroxyphenyl)retinamide
Aβ	Beta-amyloid peptide
ABTS	2,2′-azino-bis(3-ethylbenzothiazoline-6-sulfonic acid)
AD	Alzheimer’s disease
AIF	Apoptosis-inducing-factor
AIN93G	American Institute of Nutrition 93 for growth
aNF	Naphtoflavone
APG	Apigenin
ApoE−/−	ApoE knockout
ASK1	Apoptosis signal-regulating kinase 1
ATF4	Activating Transcription Factor 4
ATF6α	Transcription factor alpha
ATRA	Alpha trans-retinoic acid
BAX	Bcl-2 Associated X-protein
BBB	Blood brain barrier
Bcl-2	B-cell leukemia/lymphoma 2 protein
*Bcl-2*	*B-cell lymphoma 2*
BDNF	Brain-derived neurotrophic factor
BECN-1	Beclin-1
BFA	brefeldin A
BID	BH3 Interacting Domain Death Agonist
BiP	Binding immunoglobulin protein
BIRC	Baculoviral inhibitors of apoptosis repeat containing
BMECs	brain microvascular endothelial cells
Ca2+	Calcium
CALM2	Calmodulin 2
Caspases	Cysteine-aspartic proteases
CdCl2	Cadmium chloride
CDR1as	Cerebellum Degeneration-Related antigen 1
CHOP	C/EBP-homologous protein
COX2	Cyclooxygenase 2
COX-2	Cyclooxygenase-2
CRE	Carbapenem-resistant Enterobacterales
CREB	cAMP-response element binding protein
CSVD	Cerebral Small Vessel Disease
Cu	Copper
DCF	Dichlorofluorescein
DN	Diabetic neuropathy
DPPH	2,2-diphenyl-1-picrylhydrazyl
DRG	Dorsal root ganglion
EGC	Epigallocatechin
EGCG	Epigallocatechin-3-gallate-3-gallate
eIF2a	Eukaryotic translation initiation factor 2A
eIF2α	Initiation factor 2α
ER	Endoplasmic reticulum
ERK	Extracellular signal-regulated kinase
F11	Chrysin
F12	Kaempferol
F2	3′,4′-dihydroxyflavone
F32	Apigenin
F7	Quercetin
GADD	Growth arrest and DNA damage-inducible gene
GADD34	The growth arrest and DNA damage-inducible protein
GBM	Glioblastoma
GFAP	Glial fibrillary acidic protein
gp96	Heat shock protein glycoprotein 96 kDa
GRP78	The 78-kDa glucose-regulated protein
GRP94	Glucose-regulated protein 94
GSH/GSSG	Glutathione/Oxidized Glutathione
GSH	*Glutathione*
GSH-Px	Plasma glutathione peroxidase
GSK3β	Glycogen synthase kinase 3β
GST	Genistein
H/R	Reoxygenation
HBMECs	Human brain microvascular endothelial cells
HEK293 cells	Human embryonic kidney 293 cells
HNK	Honokiol
HSPA5	Heat Shock Protein Family A (Hsp70) Member 5
i.p	Intraperitoneally
IBMX	3-isobutyl-1-methylxanthine
IBS	Irritable bowel syndrome
ID2	Inhibitor of DNA Binding 2
IL1β	Interleukin 1 beta
IL-6	Interleukin 6
iNOS	Inducible nitric oxide synthase
IP3R	1,4,5-triphosphate receptor
IRE1a	Inositol-requiring Enzyme 1
IRE1α	Inositol-requiring transmembrane kinase/endoribonuclease 1α
IRE1α	Inositol-requiring enzyme 1 alpha
ISR	Integrated stress response
ISRIB	Integrated stress response inhibitor
JNK1	Mitogen-activated protein kinase 8
Keap1	Kelch-like ECH-associated protein 1
LBs	Lewy bodies
LC-3	Light chain 3
LPS	Lipopolysaccharide
LQ	Liquiritin
MAP	Mitogen-activated protein
MAPKs	Mitogen-activated protein kinases
MCU	Mitochondrial uniporter
*MDA*	Malondialdehyde
MDD	Major depressive disorder
MDM2	Mouse double minute 2 homolog
MEK	Mitogen-activated protein kinase
MMP	Mitochondrial membrane potential
MMP-2	Matrix metalloproteinase-2
MPP+	1-methyl-4-phenylpyridinium
MPP1	MAGUK P55 Scaffold Protein 1
MPTP	1-methyl-4-phenyl-1,2,3,6-tetrahydropyridine
mtPTP	Mitochondrial permeability transition pore
NAFLD	Non-alcoholic fatty liver disease
NF-κB	Nuclear factor-kappa B
NO	Nitric oxide
Nrf2	Nuclear factor erythroid 2-related factor 2
PA	Palmitate
PARP	Poly-ADP ribose polymerase
PARP-1	Poly (ADP-ribose) polymerase 1
PC12 cells	Rat pheochromocytoma cells
PCs	Proanthocyanidins
PD	Parkinson disease
PD	Parkinson’s disease
p-eIF2α	Eukaryotic initiation factor-2α
PERK	PKR-like endoplasmic reticulum kinase
PKA	Protein kinase A
poly Q	Polyglutamine
QcSNPs	Quercetin-modified sulfur nanoparticles
RH	Rutin hydrate
RNAi	RNA-interference
ROS	Reactive oxygen species
SCI	Spinal cord injury
SIRT1	Sirtuin 1
SOD	Superoxide dismutase
TBI	Traumatic brain injury
TG	Thapsigargin
TMD	Temporomandibular disorders
TNF-α	Tumor necrosis factor alpha
TRAF2	TNF Receptor Associated Factor 2
TUN	Tunicamycin
UPR	Unfolded protein response
WT	Wild-type C57BL/6
XBP1	X-box-binding protein 1
YKT6	YKT6 V-SNARE Homolog
ΔΨm	Mitochondrial membrane potential
