# Supplementary material for: Neuroprotective effects of flavonoids: endoplasmic reticulum as the target
Source: Front Neurosci. 2024 Jun 18;18:1348151. doi: 10.3389/fnins.2024.1348151 (PMC11218733; doi:10.3389/fnins.2024.1348151)
Supplement: Supplementary file 1 [file Table_1.docx]

Table 1: A summary of the studies on the effect of flavonoids in the treatment of age-related neurological diseases through inhibiting ER stress.

| **Chemical structure** | **Mechanisms of neuroprotection** | **Route of administration** | **Dose** | **Duration** | **Model of the disease** | **Type of study** | **Flavonoid** | **Year** | **Author(reference)** |
| --- | --- | --- | --- | --- | --- | --- | --- | --- | --- |
| 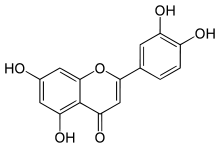 | -inhibited astrocyte overactivation (GFAP) and neuroinﬂammation (TNF-α, IL1β, IL-6, NO, COX-2, and iNOS protein)  -decreased the expression of ER stress markers GRP78 and IRE1α | Intraperitoneal injection | luteolin (20, 40mg·kg−1 ·d−1, ip) | 3 weeks | triple-transgenic mouse model of AD (3×Tg-AD) | In vivo | Luteolin | 2021 | Kou et al.(1) |
| 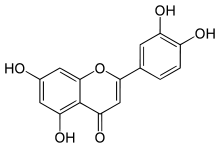 | -induced apoptosis in human glioblastoma cancer cells  -increased intracellular ROS levels  -induced expression of ER stress-associated proteins | Cell culture,  Intrap  eritoneal injections | DMSO or luteolin (10  mg/kg) | thrice per week for 35 days (15 times in total) | U251MG and U87MG human glioblastoma cell lines, male BALB/c athymic nude mice (Charles River Breeding Laboratories, Wilmington, MA) | in vitro and in vivo | Luteolin | 2017 | Wang et al.(2) |
| 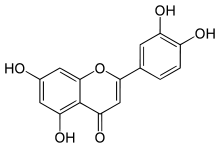 | -induced activation of caspases (caspase-12, -9, and -3)  -regulated the expression of bcl-2 family proteins  -induced expression and activation of ER stress-associated proteins | Cell culture | 10 µg |  | Neuro-2a  Mouse neuroblastoma cells | In vitro | luteolin | 2011 | Choi et al(3) |
| 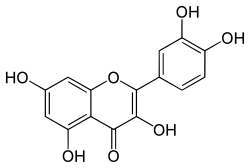 | -reduced eIF2a phosphorylation and ATF4 expression through GADD34 inductioned | oral | group 1 was fed an AIN93G diet containing 20% casein (Basal) and group 2 was fed on an AIN93G diet containing 20% casein and 0.5% quercetin (Basal þ Q).  4 g of food per day for each mouse | - | APP23 AD model mice | In vivo | Quercetin | 2015 | Hayakawa et al.(4) |
| 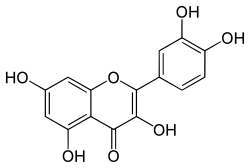 | -reduced the content of amyloid plaques  -increased in Nissl bodies  -reduced ER stress levels | injection | Quercetin (5 mg/kg), SNPs-MB (5 mg/kg), (NPs=5 mg/kg) and equal volume saline | twice a week for 5 weeks | C57BL/6 AD mice | In vivo | Quercetin | 2020 | Liu et al.(5) |
| 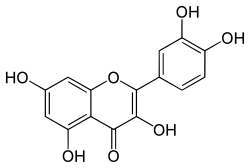 | -increased in BiP,and induction of GADD34 mRNA,suppression  of ATF4 expression.  -decreased amyloid-b. controlling ER stress. | Cell culture  were injected intraperitoneally | 25 μM quercetin,  tunicamycin at a dose of 1 lg/g body weight | 12 h | HEK293 cells,  C57BL/6J male mice | In vitro  And In vivo | Quercetin | 2011 | Ohta et al.(6) |
| 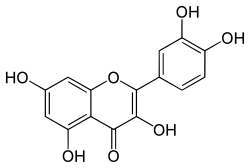 | -dieldrin-induced ER stress markers  -repressed apoptotic cell death | Cell culture | 10, 20, 40μM | 1 h | dopaminergic SN4741 cells | In vitro | Quercetin | 2016 | Park et al.(7) |
| 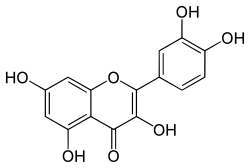 | -induced cell death and organelle stress  -caused Ca^2+^ homeostasis disruption  -induced lysosomal defects | Cell culture | - | - | glioma cells | In vitro | Quercetin | 2020 | Jang et al. (8) |
| 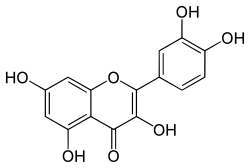 | - mobilized Ca^2+^ from intracellular stores  - inhibited Ca2+ signaling triggered by ATP  - inhibited NO release triggered by ATP | Cell culture | 1, 3, 10, and 30 μM | 1000s | bEnd.3 cells | In vitro | Quercetin | 2020 | Chen et al. (9) |
| 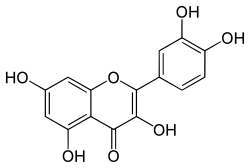 | -reversed the adverse effects of copper on cells  -reversed the increase in ROS levels  -reduced the nuclei morphological deformities  -induced autophagy  -increased ER homeostasis | Cell culture | - | - | Human neuroblastoma SH-SY5Y secondary cell lines | In vitro | Quercetin | 2022 | Chakraborty et al. (10) |
| 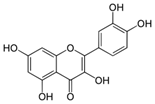 | -increased viability of the cells in radiation0induced cytotoxicity  -reduced cell inflammation and ER stress  -inhibited ER stress-induced apoptosis | Cell culture | 5‐100 μM | 24 h | dorsal root ganglia neurons | In vitro | Quercetin | 2019 | Chatterjee et al. (11) |
| 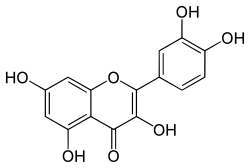 | -increased Nrf2 level  -reduced NF-κB levels  -upregulated FOXO1A, AKT1, and AKT2 genes  -regulated Akt pathway  -protected cells against vincristine-induced cytotoxicity. | Oral | 25, 50 mg/kg | between days 1-6 and 9-14 | male Sprague Dawley rats | In vivo | Quercetin | 2020 | Yardim et al. (12) |
| 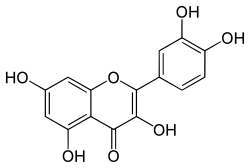 | -increased cell viability  -increased cell concentration  -inhibited apoptosis  -reduced ICAM-1 levels  -reduced oxidative stress damage  -regulated Keap1/Nrf2 and ATF6/GRP78 Proteins  -maintained blood brain barrier integrity | Cell culture | 0.1, 0.5, 1, 2, 5, 10 μmol/L | 12 h | human brain microvascular endothelial cells | In vitro | Quercetin | 2021 | Li et al. (13) |
| 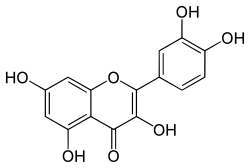 | -Prevented of AD,  -prevented A_1–42 aggregation and paralysis in the human sample |  | 1-100 μL | 48h | 24 h young adult CL2006 nematodes | Study on nematodes | Quercetin | 2014 | Regitz et al(14) |
| 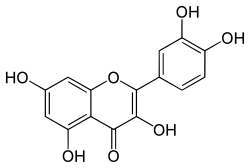 | -decreased the ER stress with modifying UPR signaling  (ER stress contributes to the pathogenesis of obesity and diabetes, which are risk factors for AD) | injective | 10-100 μL |  | Adult C57BL/6J male mice | In vivo | Quercetin | 2011 | Ohta et al(15) |
| 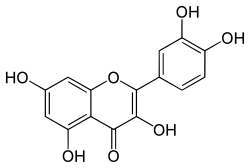 | -downregulated Hsp70 expression  - prevented the upregulation of IRE1α and BiP | tunicamycin (TN) 1 μM or thapsigargin 200 nM | tunicamycin (TN) 1 μM or thapsigargin (TG) 200 nM | 24h | human monoblastic leukemia U937 cells | both in vivo and in vitro | Quercetin | 2015 | Storniolo et al.(16) |
| 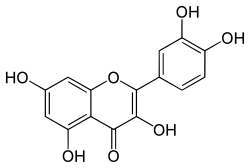 | -alleviated ethanol-induced release of liver-specific aminotransferases and lipid disorder and exhibits favorable hepatoprotective effect against ethanol hepatotoxicity by counteracting oxidative stress in vivo and in vitro. | Chronic alcohol administration for adult male rats (4.0 g/kg for 90 days) | 4.0 g/kg | 90 days | liver samples slices | In vivo | Quercetin | 2021 | Tang et al .(17) |
| 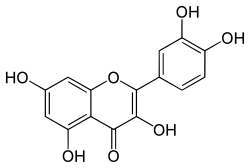 | -prevented dieldrin (20 μM)(Dieldrin is a risk factor for PD)  - induced cytotoxicity in SN4741 cells.  -suppressed the ER stress-  CHOP pathway and dieldrin-induced apoptosis in  dopaminergic neurons  -suppressed dieldrin-induced apoptotic characteristics,  including nuclear condensation, DNA fragmentation, and caspase-3/7 activation. |  | 10–40 μM | 48h | SN4741 cells | In vitro | Quercetin | 2016 | Park et al, (18) |
| 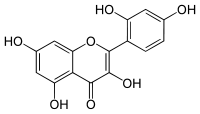 | -reduced MPP1-induced astroglial activation and nuclear translocation of nuclear factor-jB in primary cultured astrocytes | intraperitoneally (i.p.) | Morin (5 or 50 mg/kg of body weight, dissolved in phosphate-buffered saline [PBS] containing 5% ethanol) | for 12 consecutive days | acute MPTP-induced mouse PD model | In vivo | Morin | 2016 | Lee et al.(19) |
| 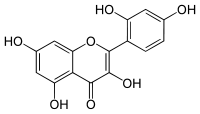 | morin significantly attenuated the loss of cell viability and apoptosis  also attenuated ROS | Cell culture, and  Intrap  eritoneal injections | Morin was given at 5, 20, 40, or 100 mg/kg body weight as daily | - | PC12 cells, and  (MPTP) mouse model of PD | in vitro and in vivo | Morin | 2010 | Zhang et al.(20) |
| 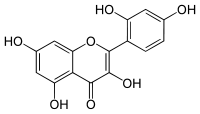 | -improved neuro behavorial score  -reduced lipid peroxidation  -increase antioxidant levels  -caused Caspase-3 and Bax downregulation and Caspase-3 and Bax upregulation  -inhibited profile of cytokine mRNA expression | Oral | 30 mg/kg b.wt | 7 days on daily basis | Male Adult Sprague-Dawley rats | In vivo | Morin | 2017 | Chen et al. (21) |
| 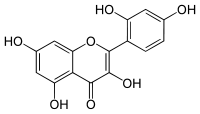 | -enhanced the nerve function  -reduced allodynia and hyperalgesia  -reduced cellular ROS  -increased Nrf2-mediated antioxidant responce | Oral | 50 and 100 mg/kg | 2 weeks | Male Sprague-Dawley rats and N2A cells | In vivo and in vitro | Morin | 2018 | Bachewal et al. (22) |
| 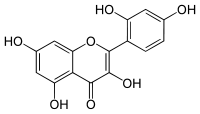 | -exhibited neuroprotective effects by modulating mitochondrial dysfunction and ROS formation | injective | 0, 1, 10, or 100 lM) | 6h | 1-methyl-4-phenyl-1,2,3,6- tetrahydropyridine (MPTP)-induced mouse model of PD | In vivo | Morin | 2016 | lee et al(23) |
| 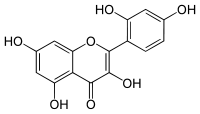 | -reduced oxidative stress. | injective | 5, 20, 40,  or 100 mg/kg |  | mouse model of PD | In vivo | Morin | 2010 | Zhang et al(24) |
| 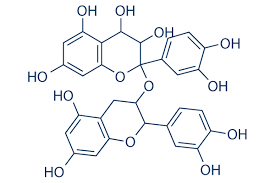 | -attenuated locomotor damages caused by lead exposure  Inhibited oxidative stress  -inhibited tau and Aβ activity in the brains od mice exposed to lead  - reduced the NF-κB activity  - inhibited IRE1/JNK-mediated and PREK/eIF2a-mediated inflammatory signaling in rats hippocampus | Oral | 100 and 200 mg/kg | 75 days | Male Wistar rats | In vivo | Proanthocyanidins | 2014 | Liu et al. (25) |
| - | - improved memory impairment  -attenuated injury in cortical neurons  -enhanced hippocampal lactate dehydrogenase Ca2+-ATPase, and Na^+^-K^+^-ATPase activity  -enhanced cortical superoxide dismutase activity  -inhibited cortical malonaldehyde expression | Intragastric perfusion | 35, 70 and 140 mg/kg | 13 days | Sprague-Dawley rats | In vivo | Flavonoids from Scutellaria baicalensis Georgi | 2013 | Shang et al. (26) |
| 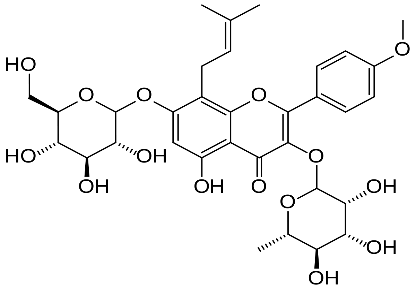 | -inhibited TNF-α, IL-1β, IL-6  -suppressed IRE1α/XBP1 pathway  -enhanced cell viability  -reduced ER stress | Cell cultrue | 0.25, 0.5, 1 mg/L | 1 h | Microglia and cortical neurons of neonatal Sprague-Dawley rats | In vitro | Icariin | 2020 | Mo et al. (27) |
|  | -reduced ER stress  --improved cognitive deficit  - prevented obesity  -inhibited JNK1 activation  -reduced hippocampal ROS levels | Oral | 150 mg/kg/day | 20 weeks | C57BL/6 male mice | In vivo | Troxerutin | 2011 | Lu et al. (28) |
| 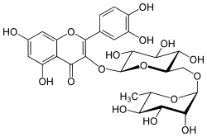 | -maintained Ca2+ homeostasis,  -inhibit ER stress, and protected mitochondria | Cell culture | 25 μM, 50 μM, or 100 μM | 4 h | MPP+-treated SH-SY5Y neuroblastoma cells, a PD cell model | In vitro | Rutin | 2019 | Enogieru et al.(29) |
| 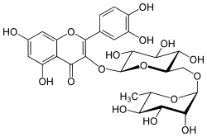  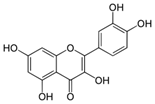  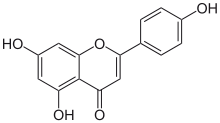  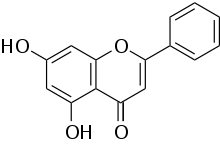  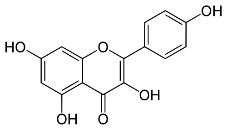  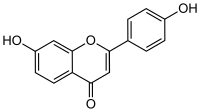 | -decreased the number of  viable cells and the mitochondrial metabolism.  -damaged  mitochondria and rough ER, inducing apoptosis  - induced a delay in cell migration, reduction on metalloproteinase (MMP-2)  expression and activity  -increased in intra- and extracellular  expression of fibronectin, and intracellular expression of laminin | cell cultures | (50µM) | 48 hours | human glioblastoma  cell lines GL-15, U251 and TG-1 cell | in vitro | rutin, quercetin (F7), apigenin (F32),  chrysin (F11), kaempferol (F12), and 3',4'-dihydroxyflavone (F2) | 2015 | Santos et al.(30) |
| 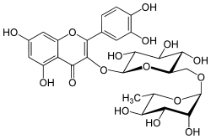 | -increased SOD and GSH_Px activities  -increased GSH levels  -lowered GSSG and MDA levels in mice braines  -inhibited brain mtPTP  -enhanced mitochondrial coupling and inhibited release of mitochondrial cytochrome C | Oral | 1 ml of rutin hydrate (100 mg/kg/day) | Daily for 30 days | Adult male Sprague-Dawley rats (9 weeks, 170–180 g) | In vivo | Rutin hydrate | 2019 | Mostafa et al. (31) |
| 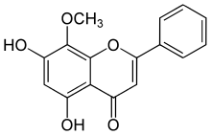 | -induced caspase-9 and caspase-3 activation,and up-regulation of cleaved PARP expression  -increased a number of signature ER stress markers | Cell culture | (25 μM) | 24 h | U87 and U251 cells originated from a human brain glioma | in vitro | Wogonin | 2012 | Tsai et al (32) |
| 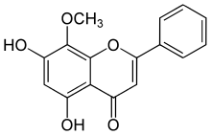 | -increased cell viability  -reduced Bax and Bcl-2 ratio  -reduced expressions of GRP78 and CHOP  -reduced p-PERK, p-eIF2α, and ATF4 levels | Cell culture | 10, 25, 50, 75, and 100 μM | 24 h | Dorsal Root Ganglion Neurons harvested from Sprague-Dawley rats | In vitro | Wogonin | 2015 | Chen et al. (33) |
| 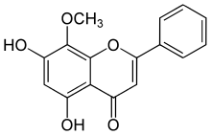 | -promoted apoptosis  -promoted the release of cytochrome c  - altered the expression of certain members of Bcl-2 family (Bcl-2, Bax and Bid)  -increased the activation of caspase-3, caspase-8, caspase-9, and PARP-1  -mitochondrial dysfunction  -induced the expression of ER stress-related proteins (GRP78/Bip and GRP94/ gp96)  -activation of caspase-12 and caspase-4  -increase the expression of IRE1α and TRAF2, and phosphorylation of ASK1 | Cell culture | 0-150μM |  | Two malignant neuroblastoma cell lines (SK-N-BE2 and IMR-32cells) | In vitro | Wogonin | 2015 | Ge et al(34) |
| 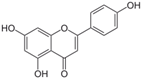  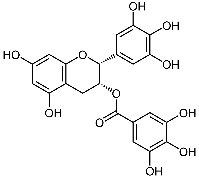  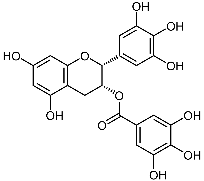  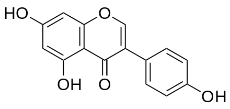 | -Induced ROS,  -Activated JNK1  -Suppressed Expression of Antiapoptotic and Inflammatory Proteins. | Cell Culture | 50 lM | 0.5 hours to 24 hours (30-1440 minutes) | human glioblastoma T98G and U87MG cells | in vitro | apigenin,  epigallocatechin, (-)-epigallocatechin-3-gallate (EGCG), and genistein | 2010 | Das et al.(35) |
| 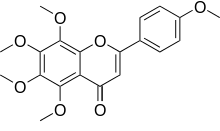  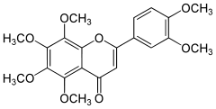  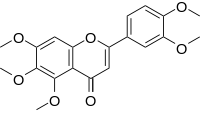 | -methoxyﬂavones mildly activated the eukaryotic initiation factor 2$\alpha$ and nuclear factor erythroid 2-related factor pathways, regulation of ER stress | intraperitoneally injected | IN19 (10 mg.kg.day)in saline, including 10% Cremophore EL (Sigma) and 10% DMSO, or with the dissolving solution (vehicle) | daily for 2 or 4 days | C57BL/6 mice | In vivo | IN19 (tangeretin: 5,6,7,8,4΄-pentamethoxyﬂavone), IN69 (nobiletin: 5,6,7,8,3΄,4΄-hexamethoxyﬂavone), IN72 (5,6,7,4΄tetramethoxyﬂavone), and IN88 (sinensetin: 5,6,7,3΄,4΄-pentamethoxyﬂavone) | 2007 | Takano et al(36) . |
| 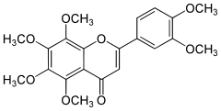  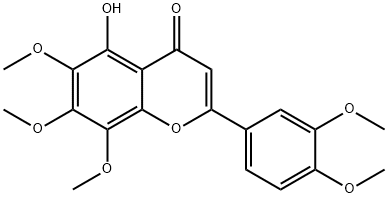  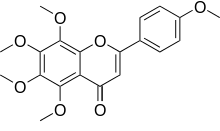  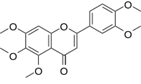  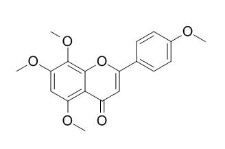 | -potently enhanced CRE-dependent transcription and neurite outgrowth by activating ERK/MAP kinase-dependent signalling to increase CREB phosphorylation. | cell culture,  oral | 100 lM nobiletin,  Twenty-five to 200 mg/kg/day of nobiletin or  vehicle | 48-h,11 days | PC12D cells, male ddY mice | in vitro  and  In vivo | nobiletin,  5-demethylnobiletin,  tangeretin, sinensetin, 6-demethoxytangeretin,  6-demethoxynobiletin | 2005 | [Nagase](https://www.sciencedirect.com/science/article/abs/pii/S0006291X0502228X#!) et al.(37) |
| 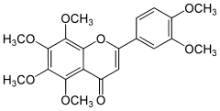 | -increased in phosphorylation of MEK and Erk/MAP kinase, stimulated CREB phosphorylation and CREmediated transcription in a MEK/Erk/MAP-kinase-dependent signaling cascade. -activated PKA activity, synergistically augumented the intracellular cAMP level and CRE-mediated transcription | cell culture | 1 µM forskolin, or 100 µM nobiletin | 5 h | PC12D cells | in vitro | Nobiletin | 2005 | Nagase et al.(38) |
| 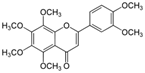  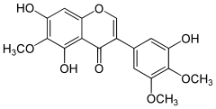 | inhibited effect on barium sulphate transport | Intrap  eritoneal injections | 50 mg/kg | - | Male DDY mice | In vivo | Nobiletin, irigenin,  pentamethyl quercetin | 1982 | Nikaido et al.(39) |
| 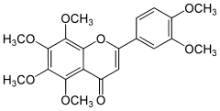 | -Increased TXNIP expression in cells treated with tunicamycin  -reduce apoptosis caused by tunicamycin | Cell culture | 100 μM | 24 h | SK-N-SH human neuroblastoma cells | In vitro | Nobiletin | 2013 | Ikeda et al. (40) |
| 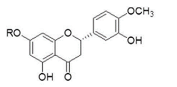 | -Elevated BDNF  -reversed  The disruptive  Effect of global cerbarl  I/R on memory | Oral |  | 13 years | Neuropharmacology | In Vitro | Cirtus (hespertin and hesperidin)  (Hst and Hsd) | 2014 | Roohbakhsh et al.(41) |
| 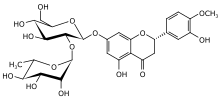 | -improved cell proliferation by reverse PDI S-nitrosylation | Cell culture | 10 mM | 24 h | SH-SY5Y cells cultured in high-glucose media | In vitro | neohesperidin | 2021 | Ogura J et al(42) |
| 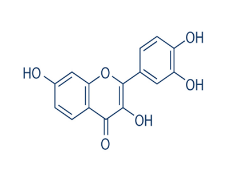 | -reduced oxidative damage and neurotoxicity,  -Increased autophagy in response to ER stress  Reducing ROSs synthesis | oral | 25 mg/kg | 3 weeks | A rat model of reserpine induced fibromyalgia | In vivo | Fisetin | 2021 | Ghoneim FM et al. (43) |
| 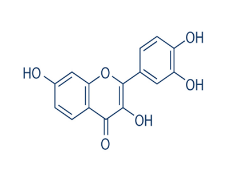 | -induced protection from cell death  -inhibited expression of apoptotic and autophagic proteins  -inhibited endoplasmic reticulum stress gene expression | Cell culture | 5–20 µM | 16 hours | rat adrenal pheochromocytoma cell line PC12 | In vivo | Fisetin | 2017 | Yen et al. (44) |
| 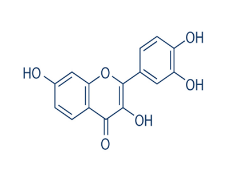 | -suppressed GRP78-mediated ER stress  - improved mitochondrial dysfunction-inhibited ROS production  -improved Nrf2 | After treatments, 10 μL of Cell Counting Kit-8 reagent was added into each well. After incubation for 4 h at 37 °C, the absorbance of each well at 450 nm was measured using a microplate reader to examine the number of viable cells. | 10 μL | 4h | human and murine hepatocytes | In vivo | Fisetin | 2022 | Dai et al. (45) |
| 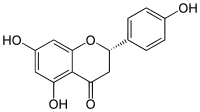 | - restored ER homeostasis, climbing capacity, and lifespan defects of ReepA mutation | oral | 0.5 mM | 12:12 h light: dark cycle | Hereditary Spastic Paraplegia -associated ReepA−541 mutation | In vivo | Naringenin | 2019 | Napoli B et al(46) |
| 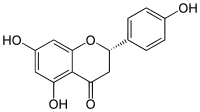 | - reduced brain edema, cell insults and neurological deficit after TBI  - reduced oxidative stress caused by TBI | intraperitoneally | 50 mg/kg and (100 mg/kg | 30 min post-TBI, and then daily for 3 or 7 days longer | ICR adult male mice (6–8 weeks, 25–30 g) | In vivo | Naringenin | 2021 | Deng et al. (47) |
| 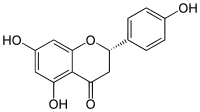 | -suppressed protein aggregation  -reduce ER stress  -enhanced the grp78 promoter activity | Cell culture | 0.05, 0.1, 0.2, 0.4, 0.6, 0.8 mM | 72 h | HeLa-tetQ97 cells, African green monkey kidney COS-7 cells, Mouse fibroblast C3H10T1/2  cells | In vitro | Naringenin | 2012 | Yamagishi et al. (48) |
| 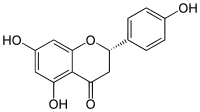 | -increased in DPPH and ABTS radical scavenging activity, inhibition of Conca-navalinA (ConA)-induced activation of T cell proliferation and NO or ROS production in PS-induced RAW264.7 macrophage cells and cause Antioxidant activity | Cell culture | 500ng/ml | 24hr | Human hepatoma cell line HepG2, mouse colon cancer cell line CT26, mouse melanoma cell line B16F1, and mouse peritoneal macrophage RAW264.7 | In vitro | Kaempferol | 2018 | Wang J et al(49) |
| 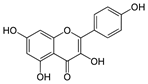 | -Decreased ER stress by inhibiting expiration of GRP78 ( a chaperon) and CHOP( ER stress-associated pro-apoptotic transcription factor) | Cell culture | 50 µM | - | human neuroblastoma cell line IMR32 | In vitro | kaempferol | 2018 | Abdullah A et al(50) |
| 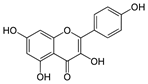 | -induced differentiation of neuroblastoma cells via the IRE1a-XBP1 pathway | Cell culture | 50 µM | 4 days | IMR32 and Neuro2a cell lines | In vitro | kaempferol | 2019 | Abdullah A et al(51) |
| 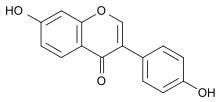  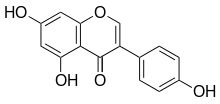 | -prevented the cytotoxicity induced by homocysteine  -inhibited apoptosis induced by homocysteine  -inhibited the ER stress induced by homocysteine  -proinhibited the DNA damage induced by homocysteine | Cell culture | 10-8 M | 24h | Human neuroblastoma  SH-SY5Y cells | In vitro | daidzein and genistein | 2010 | Park et al.(52) |
| 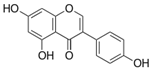 | -protected against cell death via estrogen receptor-dependent pathways,  - suppressed ER stress as determined by decreased expressions of the BiP mRNA, spliced X-box binding protein-1 mRNAs, and C/EBP homologous protein  -TM activated glycogen synthase kinase 3beta, a kinase involved in tau phosphorylation; in contrast, isoflavones inactivated GSK3beta and decreased tau hyperphosphorylation | Cell culture | 50nM | 48h | SH-SY5Y human neuroblastoma cells | In vitro | genistein | 2008 | Park et al. (53) |
| 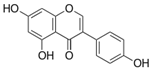 | -reduced body fat  -reduced systemic resistance in mice  -reduced active form of JNK and endoplasmic reticulum stress  -reduced Aβ protein deposition  -alleviated tau phosphorylation | oral | High fat diet containing 0.05% genistein | 24 weeks | C57BL/6 mice and ApoE^−/−^ mice | In vivo | Genistein | 2016 | Park et al. (54) |
| 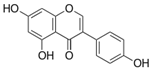 | -reduced cell viability, subG1 accumulation, -increased caspase-3 activity  -down regulated Id2 to induce differentiation,  -increased pro-apoptotic Bax and decreased anti-apoptotic Bcl-2,  -down regulated the baculovirus inhibitor-of-apoptosis repeat containing (BIRC) proteins such as BIRC-2 and BIRC-3, and activation of calpain and caspase-3 | Cell culture | a daily dose of 4-HPR (20 μg/kg/day), GST (2 mg/kg/day), or 4-HPR (20 μg/kg/day) + 4 h later GST (2 mg/kg/ day) for 8 days. |  | human malignant neuroblastoma SH-SY5Y and SK-N-BE2 cells | In vitro | genistein | 2011 | Karmakar et al(55) |
| 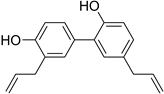  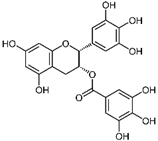 | -induced ER stress and UPR as expected from its ability to interact with GRP78 | Cell culture | 0.187 mmol | 24h | SH-SY5Y and NGP human neuroblastoma cell lines | In vitro | honokiol (HNK) and Epigallocatechin gallate (EGCG) | 2013 | Martin et al. (56) |
| 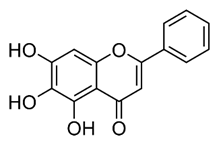 | -abated thapsigargin (TG),  -reduced the TG- and BFA-triggered explanation of ER stress-linked proteins, including CHOP  -Provoked proapoptotic activity through ROS-initiated and Ca +2-dependent mitochondrial dysfunction pathways in various cell types |  |  |  |  |  | Baicalein | 2014 | Obulesu et al.(57) |
| 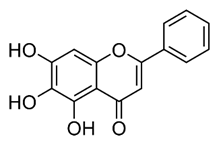 | -protected neuronal cells from cell death  -inhibited caspase-12/-3 and poly(ADP-ribose) polymerase cleavage  -regulated unfolded protein responses  -inhibited MAPKs activations  -inhibited ROS accumulation in neuronal cells | Cell culture | 10,25, and 50 μM | 1h | murine hippocampal neuronal cell line HT22 | In vitro | Baicalein | 2010 | Choi et al. (58) |
| 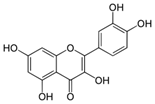  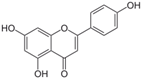  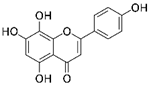  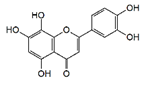 | -activated cellular relocalization of p53 and DNA fragmentation-dependent cell death  -Overexpressed genes related to cellular stress, protein synthesis, cell survival and death, ER stress inducer and sensor HSPA5 and other ER stress-related genes CALM2 and YKT6 | Cell culture | 10 µg/mL |  | human neuroblastoma SH-SY5Y cells. | In vitro | glycosides of quercetin, apigenin, isoscutellarein and hypolaetin | 2016 | lantto et al(59) |
| 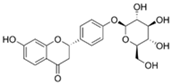 | - inhibited ER stress  -maintained the integrity of  BBB  -Promoted cell proliferation, angiogenesis, and migration  - reduced  MMP damage and apoptosis  - decreased the expression of related adhesion molecules  - reduced the production of  reactive oxygen species  - activated Keap1/Nrf2 antioxidant pathway | Cell culture | 0.1-10 μmol/L | 8-12 h | Human Brain Microvascular Endothelial Cells (HBMECs) injured by hypoxia reoxygenation | In vitro | Liquiritin | 2021 | Li et al (60) |
|  | -protected PC12 cells against various oxidative stressors such as hydrogen peroxide, linoleic acid,  - reversed hydroperoxide or tert-butyl hydroperoxide and α-ZOL and β-ZOL-induced cytotoxicity | HCT116 cells were treated for 24 h with increasing concentrations (100–400 μM) of α-ZOL and β-ZOL and cell viability determined by FDA assay. | 100–400 μM | 24h | HCT116 cells | In vivo | α-ZOL or β-ZOL combined with Quercetin (QUER) | 2016 | Ben Salem et al. (61) |
|  | -ameliorated cytotoxicity caused by H_2_O_2_  -inhibited ER stress  -activated Nrf2 Signaling | 100 μmol/L Baicalin pretreatment and 4 h of H2O2 stimulation | 100 μmol/L Baicalin pretreatment | 4h | Human renal proximal tubular epithelial cell line HK-2 cells | in vitro | Baicalin | 2014 | Lin et al. (62) |
|  | -Increased Sestrin2 expressio  -activated ER stress in HepG2 cells | cells were treated with BV (20 mM) and TM (1 mM) for 12h to detect mRNA and protein analysis | cells were treated with BV (20 mM) and TM (1 mM) | 12h | HepG2 cells | mechanistic study | Bavachin | 2021 | Yang et al. (63) |

1. Kou J-j, Shi J-z, He Y-y, Hao J-j, Zhang H-y, Luo D-m, et al. Luteolin alleviates cognitive impairment in Alzheimer’s disease mouse model via inhibiting endoplasmic reticulum stress-dependent neuroinflammation. Acta Pharmacologica Sinica. 2022;43(4):840-9.

2. Wang Q, Wang H, Jia Y, Pan H, Ding H. Luteolin induces apoptosis by ROS/ER stress and mitochondrial dysfunction in gliomablastoma. Cancer chemotherapy and pharmacology. 2017;79(5):1031-41.

3. Choi AY, Choi JH, Yoon H, Hwang K-Y, Noh MH, Choe W, et al. Luteolin induces apoptosis through endoplasmic reticulum stress and mitochondrial dysfunction in Neuro-2a mouse neuroblastoma cells. European journal of pharmacology. 2011;668(1-2):115-26.

4. Hayakawa M, Itoh M, Ohta K, Li S, Ueda M, Wang M-x, et al. Quercetin reduces eIF2α phosphorylation by GADD34 induction. Neurobiology of Aging. 2015;36(9):2509-18.

5. Liu Y, Gong Y, Xie W, Huang A, Yuan X, Zhou H, et al. Microbubbles in combination with focused ultrasound for the delivery of quercetin-modified sulfur nanoparticles through the blood brain barrier into the brain parenchyma and relief of endoplasmic reticulum stress to treat Alzheimer's disease. Nanoscale. 2020;12(11):6498-511.

6. Ohta K, Mizuno A, Li S, Itoh M, Ueda M, Ohta E, et al. Endoplasmic reticulum stress enhances γ-secretase activity. Biochemical and Biophysical Research Communications. 2011;416(3):362-6.

7. Park E, Chun HS. Protective effects of quercetin on dieldrin-induced endoplasmic reticulum stress and apoptosis in dopaminergic neuronal cells. Neuroreport. 2016;27(15):1140-6.

8. Jang E, Kim IY, Kim H, Lee DM, Seo DY, Lee JA, et al. Quercetin and chloroquine synergistically kill glioma cells by inducing organelle stress and disrupting Ca2+ homeostasis. 2020;178:114098.

9. Chen CY, Hour MJ, Shiao LR, Wong KL, Leung YM, Chan P, et al. Quercetin depletes intracellular Ca2+ stores and blunts ATP‐triggered Ca2+ signaling in bEnd. 3 endothelial cells. 2020;34(2):213-21.

10. Chakraborty J, Pakrashi S, Sarbajna A, Dutta M, Bandyopadhyay JJBTER. Quercetin Attenuates Copper-Induced Apoptotic Cell Death and Endoplasmic Reticulum Stress in SH-SY5Y Cells by Autophagic Modulation. 2022:1-20.

11. Chatterjee J, Langhnoja J, Pillai PP, Mustak MSJJoB, Toxicology M. Neuroprotective effect of quercetin against radiation‐induced endoplasmic reticulum stress in neurons. 2019;33(2):e22242.

12. Yardim A, Kandemir FM, Ozdemir S, Kucukler S, Comakli S, Gur C, et al. Quercetin provides protection against the peripheral nerve damage caused by vincristine in rats by suppressing caspase 3, NF-κB, ATF-6 pathways and activating Nrf2, Akt pathways. 2020;81:137-46.

13. Li M-T, Ke J, Guo S-F, Wu Y, Bian Y-F, Shan L-L, et al. The protective effect of quercetin on endothelial cells injured by hypoxia and reoxygenation. 2021:2893.

14. Regitz. C, Dußling. LM, Wenzel U. Amyloid-beta (Aβ₁₋₄₂)-induced paralysis in Caenorhabditis elegans is inhibited by the polyphenol quercetin through activation of protein degradation pathways. Mol Nutr Food Res. 2014;58(10):1931-40.

15. Ohta. K, Mizuno. A, Li. S, Itoh. M, Ueda. M, Ohta. E, et al. Apoptosis in Alzheimer's disease: an understanding of the physiology, pathology and therapeutic avenues. Neurochem Res. 2014;39(12):2301-12.

16. Storniolo A, Raciti M, Cucina A, Bizzarri M, Di Renzo L. Quercetin Affects Hsp70/IRE1<i>α</i> Mediated Protection from Death Induced by Endoplasmic Reticulum Stress. Oxidative Medicine and Cellular Longevity. 2015;2015:645157.

17. Tang Y, Gao C, Xing M, Li Y, Zhu L, Wang D, et al. Quercetin prevents ethanol-induced dyslipidemia and mitochondrial oxidative damage. Food and chemical toxicology : an international journal published for the British Industrial Biological Research Association. 2012;50(5):1194-200.

18. Park. E, Chun. HS. Protective effects of quercetin on dieldrin-induced endoplasmic reticulum stress and apoptosis in dopaminergic neuronal cells. Neuroreport. 2016;27(15):1140-6.

19. Lee KM, Lee Y, Chun HJ, Kim AH, Kim JY, Lee JY, et al. Neuroprotective and anti‐inflammatory effects of morin in a murine model of Parkinson's disease. Journal of neuroscience research. 2016;94(10):865-78.

20. Zhang Z-t, Cao X-b, Xiong N, Wang H-c, Huang J-s, Sun S-g, et al. Morin exerts neuroprotective actions in Parkinson disease models in vitro and in vivo. Acta Pharmacologica Sinica. 2010;31(8):900-6.

21. Chen Y, Li Y, Xu H, Li G, Ma Y, Pang YJJAJoT, Complementary, et al. Morin mitigates oxidative stress, apoptosis and inflammation in cerebral ischemic rats. 2017;14(2):348-55.

22. Bachewal P, Gundu C, Yerra VG, Kalvala AK, Areti A, Kumar AJB. Morin exerts neuroprotection via attenuation of ROS induced oxidative damage and neuroinflammation in experimental diabetic neuropathy. 2018;44(2):109-22.

23. Kyung Moon Lee, Lee. Y, Chun. HJ, Kim. AH, Kim. JY, Lee. JY, et al. Neuroprotective and anti‐inflammatory effects of morin in a murine model of Parkinson's disease. Journal of Neuroscience Research. 2016;94(10):865-78.

24. ZHANG. Z-t, CAO. X-b, XIONG. N, WANG. H-c, HUANG. J-s, SUN. S-g, et al. Morin exerts neuroprotective actions in Parkinson disease models in vitro and in vivo. Acta Pharmacologica Sinica. 2010;31(8):900-6.

25. Liu C-M, Ma J-Q, Liu S-S, Zheng G-H, Feng Z-J, Sun J-MJF, et al. Proanthocyanidins improves lead-induced cognitive impairments by blocking endoplasmic reticulum stress and nuclear factor-κB-mediated inflammatory pathways in rats. 2014;72:295-302.

26. Shang Y, Zhang H, Cheng J, Miao H, Liu Y, Cao K, et al. Flavonoids from Scutellaria baicalensis Georgi are effective to treat cerebral ischemia/reperfusion. 2013;8(6):514.

27. Mo Z-t, Liao Y-l, Zheng J, Li W-nJLs. Icariin protects neurons from endoplasmic reticulum stress-induced apoptosis after OGD/R injury via suppressing IRE1α-XBP1 signaling pathway. 2020;255:117847.

28. Lu J, Wu D-m, Zheng Z-h, Zheng Y-l, Hu B, Zhang Z-fJB. Troxerutin protects against high cholesterol-induced cognitive deficits in mice. 2011;134(3):783-97.

29. Enogieru. AB, Haylett. WL, Miller. HC, Westhuizen. FHvd, Hiss. DC, Ekpo. OE. Attenuation of Endoplasmic Reticulum Stress, Impaired Calcium Homeostasis, and Altered Bioenergetic Functions in MPP(+)-Exposed SH-SY5Y Cells Pretreated with Rutin. Neurotox Res. 2019;36(4):764-76.

30. Santos BL, Oliveira MN, Coelho PLC, Pitanga BPS, da Silva AB, Adelita T, et al. Flavonoids suppress human glioblastoma cell growth by inhibiting cell metabolism, migration, and by regulating extracellular matrix proteins and metalloproteinases expression. Chemico-Biological Interactions. 2015;242:123-38.

31. Mostafa DG, Khaleel EF, Badi RM, Abdel-Aleem GA, Abdeen HMJNr. Rutin hydrate inhibits apoptosis in the brains of cadmium chloride-treated rats via preserving the mitochondrial integrity and inhibiting endoplasmic reticulum stress. 2019;41(7):594-608.

32. Tsai C-F, Yeh W-L, Huang SM, Tan T-W, Lu D-YJIjoms. Wogonin induces reactive oxygen species production and cell apoptosis in human glioma cancer cells. 2012;13(8):9877-92.

33. Chen F, Wu R, Zhu Z, Yin W, Xiong M, Sun J, et al. Wogonin protects rat dorsal root ganglion neurons against tunicamycin-induced ER stress through the PERK-eIF2α-ATF4 signaling pathway. 2015;55(4):995-1005.

34. Ge W, Yin Q, Xian H. Wogonin induced mitochondrial dysfunction and endoplasmic reticulum stress in human malignant neuroblastoma cells via IRE1α-dependent pathway. Journal of Molecular Neuroscience. 2015;56(3):652-62.

35. Das A, Banik NL, Ray SK. Flavonoids activated caspases for apoptosis in human glioblastoma T98G and U87MG cells but not in human normal astrocytes. Cancer: Interdisciplinary International Journal of the American Cancer Society. 2010;116(1):164-76.

36. Takano K, Tabata Y, Kitao Y, Murakami R, Suzuki H, Yamada M, et al. Methoxyflavones protect cells against endoplasmic reticulum stress and neurotoxin. American Journal of Physiology-Cell Physiology. 2007;292(1):C353-C61.

37. Nagase H, Omae N, Omori A, Nakagawasai O, Tadano T, Yokosuka A, et al. Nobiletin and its related flavonoids with CRE-dependent transcription-stimulating and neuritegenic activities. Biochemical and biophysical research communications. 2005;337(4):1330-6.

38. Nagase H, Yamakuni T, Matsuzaki K, Maruyama Y, Kasahara J, Hinohara Y, et al. Mechanism of neurotrophic action of nobiletin in PC12D cells. Biochemistry. 2005;44(42):13683-91.

39. Nikaido T, Ohmoto T, Sankawa U, Hamanaka T, Totsuka K. Inhibition of cyclic AMP phosphodiesterase by flavonoids. Planta medica. 1982;46(11):162-6.

40. Ikeda A, Nemoto K, Yoshida C, Miyata S, Mori J, Soejima S, et al. Suppressive effect of nobiletin, a citrus polymethoxyflavonoid that downregulates thioredoxin-interacting protein expression, on tunicamycin-induced apoptosis in SK-N-SH human neuroblastoma cells. 2013;549:135-9.

41. Roohbakhsh A, Parhiz H, Soltani F, Rezaee R, Iranshahi M. Neuropharmacological properties and pharmacokinetics of the citrus flavonoids hesperidin and hesperetin—A mini-review. Life sciences. 2014;113(1-2):1-6.

42. Ogura J, Sugiura H, Tanaka A, Ono S, Sato T, Sato T, et al. Glucose-induced oxidative stress leads to in S-nitrosylation of protein disulfide isomerase in neuroblastoma cells. Biochim Biophys Acta Gen Subj. 2021;1865(11):129998.

43. Ghoneim FM, Abo-Elkhair SM, Elsamanoudy AZ, Shabaan DA. Evaluation of endothelial dysfunction and autophagy in fibromyalgia-related vascular and cerebral cortical changes and the ameliorative effect of fisetin. Cells. 2021;11(1):48.

44. Yen J-H, Wu P-S, Chen S-F, Wu M-JJIjoms. Fisetin protects PC12 cells from tunicamycin-mediated cell death via reactive oxygen species scavenging and modulation of Nrf2-driven gene expression, SIRT1 and MAPK signaling in PC12 cells. 2017;18(4):852.

45. Dai X, Kuang Q, Sun Y, Xu M, Zhu L, Ge C, et al. Fisetin represses oxidative stress and mitochondrial dysfunction in NAFLD through suppressing GRP78-mediated endoplasmic reticulum (ER) stress. 2022.

46. Napoli B, Gumeni S, Forgiarini A, Fantin M, De Filippis C, Panzeri E, et al. Naringenin Ameliorates Drosophila ReepA Hereditary Spastic Paraplegia-Linked Phenotypes. Front Neurosci. 2019;13:1202.

47. Deng C, Yi R, Fei M, Li T, Han Y, Wang HJBR. Naringenin attenuates endoplasmic reticulum stress, reduces apoptosis, and improves functional recovery in experimental traumatic brain injury. 2021;1769:147591.

48. Yamagishi N, Yamamoto Y, Noda C, Hatayama TJB, Bulletin P. Naringenin inhibits the aggregation of expanded polyglutamine tract-containing protein through the induction of endoplasmic reticulum chaperone GRP78. 2012;35(10):1836-40.

49. Wang J, Fang X, Ge L, Cao F, Zhao L, Wang Z, et al. Antitumor, antioxidant and anti-inflammatory activities of kaempferol and its corresponding glycosides and the enzymatic preparation of kaempferol. PLoS One. 2018;13(5):e0197563.

50. Abdullah A, Ravanan P. Kaempferol mitigates Endoplasmic Reticulum Stress Induced Cell Death by targeting caspase 3/7. Sci Rep. 2018;8(1):2189.

51. Abdullah A, Talwar P, d'Hellencourt CL, Ravanan P. IRE1α is critical for Kaempferol-induced neuroblastoma differentiation. Febs j. 2019;286(7):1375-92.

52. Park Y-J, Jang Y, Kwon YH. Protective effect of isoflavones against homocysteine-mediated neuronal degeneration in SH-SY5Y cells. Amino Acids. 2010;39(3):785-94.

53. Park YJ, Jang YM, Kwon YH. Isoflavones prevent endoplasmic reticulum stress-mediated neuronal degeneration by inhibiting tau hyperphosphorylation in SH-SY5Y cells. J Med Food. 2009;12(3):528-35.

54. Park Y-J, Ko JW, Jeon S, Kwon YHJN. Protective effect of genistein against neuronal degeneration in ApoE−/− mice fed a high-fat diet. 2016;8(11):692.

55. Karmakar S, Choudhury SR, Banik NL, Ray SK. Induction of mitochondrial pathways and endoplasmic reticulum stress for increasing apoptosis in ectopic and orthotopic neuroblastoma xenografts. Journal of Cancer Therapy. 2011;2(02):77.

56. Martin S, Lamb HK, Brady C, Lefkove B, Bonner MY, Thompson P, et al. Inducing apoptosis of cancer cells using small-molecule plant compounds that bind to GRP78. Br J Cancer. 2013;109(2):433-43.

57. Obulesu. M, Lakshmi. MJ. Protective effects of quercetin on dieldrin-induced endoplasmic reticulum stress and apoptosis in dopaminergic neuronal cells. Neuroreport. 2016;27(15):1140-6.

58. Choi JH, Choi AY, Yoon H, Choe W, Yoon K-S, Ha J, et al. Baicalein protects HT22 murine hippocampal neuronal cells against endoplasmic reticulum stress-induced apoptosis through inhibition of reactive oxygen species production and CHOP induction. 2010;42(12):811-22.

59. Lantto TA, Laakso I, Dorman HD, Mauriala T, Hiltunen R, Kõks S, et al. Cellular stress and p53-associated apoptosis by Juniperus communis L. Berry extract treatment in the human SH-SY5Y neuroblastoma cells. International journal of molecular sciences. 2016;17(7):1113.

60. Li M, Ke J, Deng Y, Chen C, Huang Y, Bian Y, et al. The Protective Effect of Liquiritin in Hypoxia/Reoxygenation-Induced Disruption on Blood Brain Barrier. Front Pharmacol. 2021;12:671783.

61. Ben Salem I, Prola A, Boussabbeh M, Guilbert A, Bacha H, Lemaire C, et al. Activation of ER stress and apoptosis by α- and β-zearalenol in HCT116 cells, protective role of Quercetin. Neurotoxicology. 2016;53:334-42.

62. Lin M, Li L, Zhang Y, Zheng L, Xu M, Rong R, et al. Baicalin ameliorates H2O2 induced cytotoxicity in HK-2 cells through the inhibition of ER stress and the activation of Nrf2 signaling. International Journal of Molecular Sciences. 2014;15(7):12507-22.

63. Yang Y, Guo G, Zhou W, Ge Y, Fan Z, Liu Q, et al. Sestrin2 protects against bavachin induced ER stress through AMPK/mTORC1 signaling pathway in HepG2 cells. Journal of Pharmacological Sciences. 2021;145(2):175-86.
